# Supplementary material for: Kinetic and data-driven modeling of pancreatic β-cell central carbon metabolism and insulin secretion
Source: PLoS Comput Biol. 2022 Oct 17;18(10):e1010555. doi: 10.1371/journal.pcbi.1010555 (PMC9612825; doi:10.1371/journal.pcbi.1010555)

S1 Supporting Information

Figure A. Metabolomics data collected from published literature. The fold change direction (increase- blue, decrease-red, or no change-yellow) in 13 published mass spectrometry datasets (*x-axis)* for all metabolites present in the computational model.

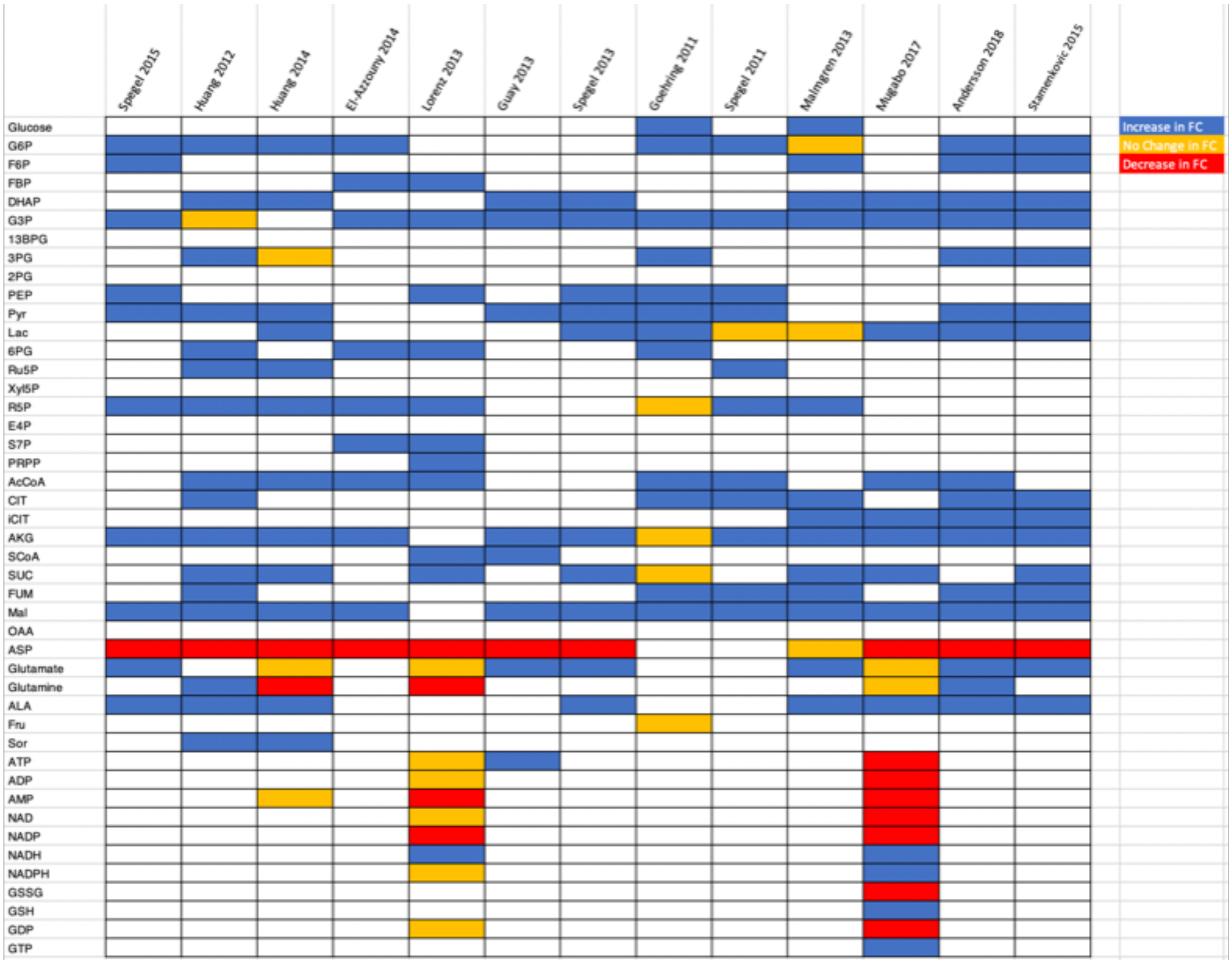

Figure B. eFAST sensitivity analysis. A global sensitivity analysis was used to identify Vmax values that significantly affect the predicted metabolite concentrations at different time points (based on experiments).

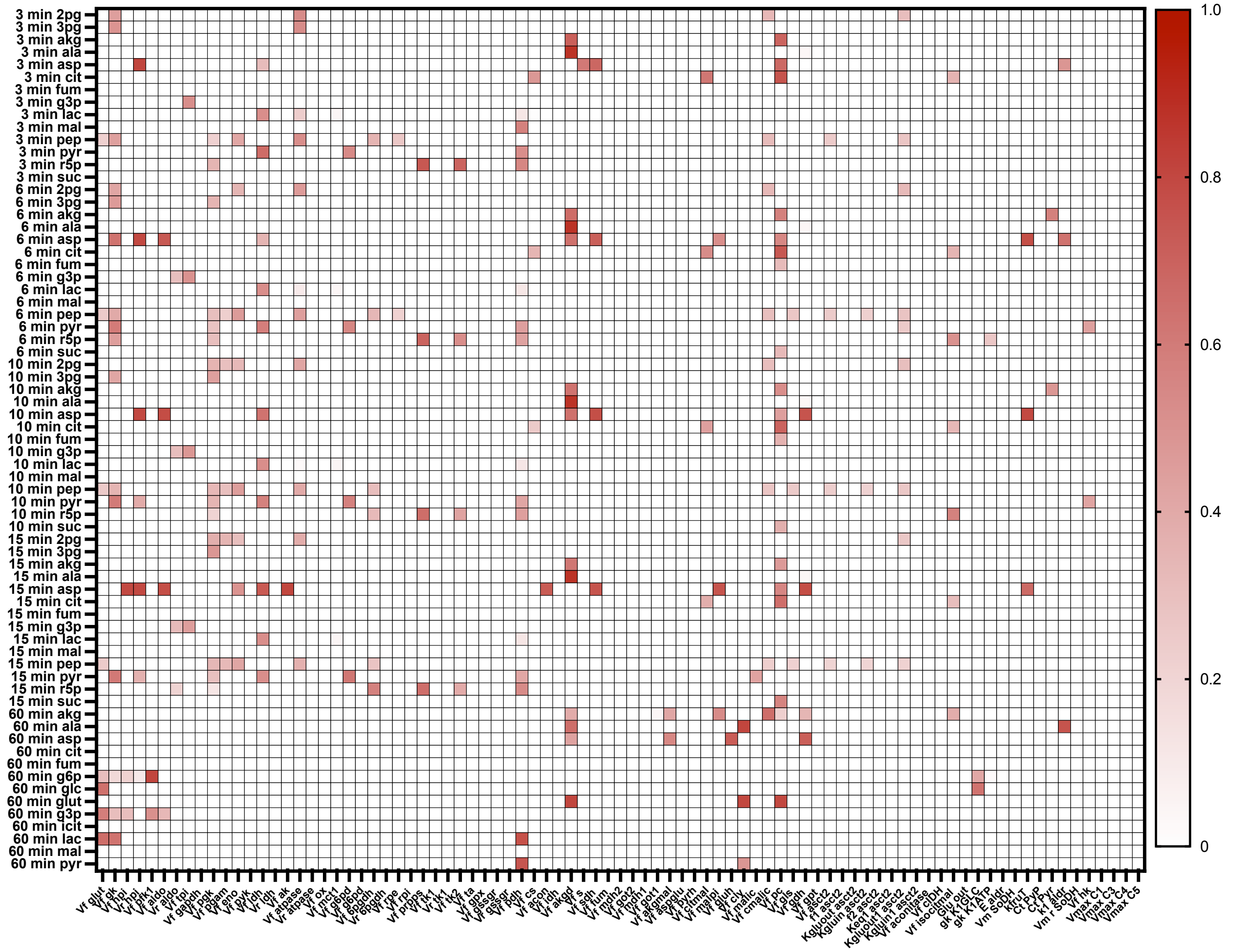

Figure C. Estimated parameter values. Having run an eFAST sensitivity analysis, 32 model parameters were found to be influential and were subsequently fit using PSO. The 8 best parameter fits were used for all subsequent analyses and for building the partial least squares regression model. The distribution of fitted model parameter values is shown here.

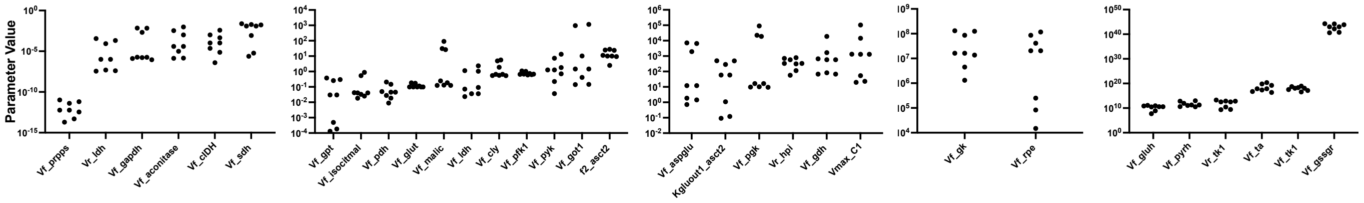

Figure D. Predicted metabolite timecourses. Intracellular metabolite amounts, simulating treatment with 16.7mM glucose. All metabolites converge to steady state concentrations. Each subplot shows the average and standard deviation of 8 distinct parameter sets.

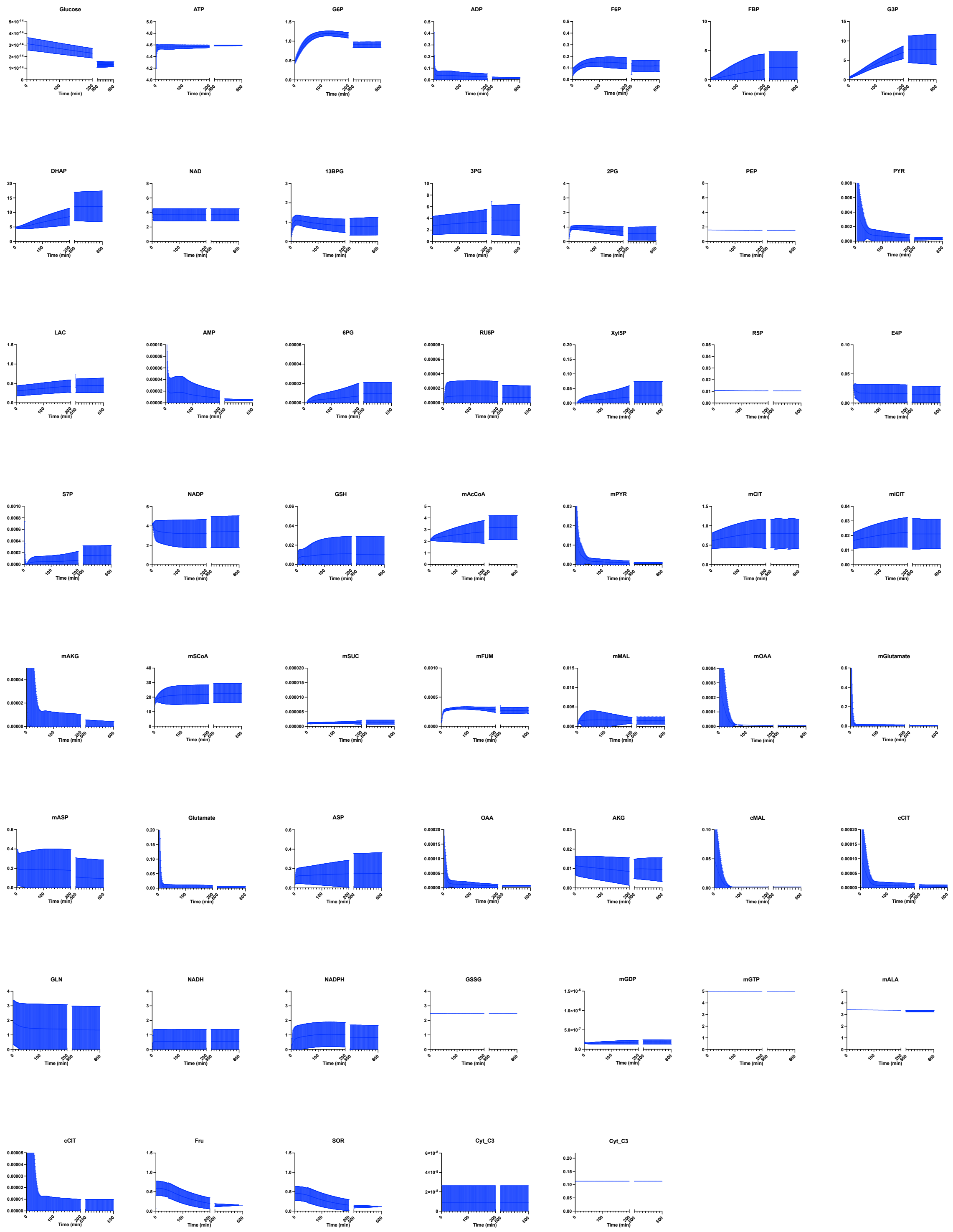

Figure E. Flux data collected from published literature. Comparison of model predictions (blue) to experimentally measured flux measurements not used in model fitting (gray).

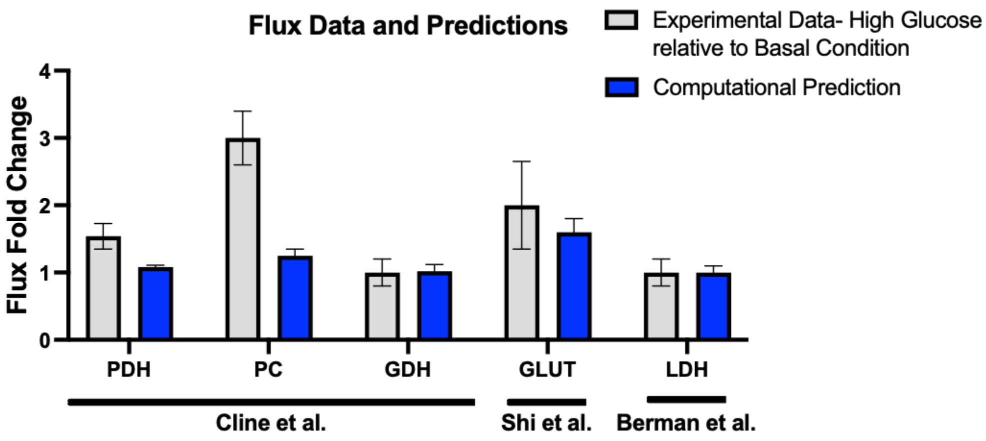

Figure F. Effect of *ak* reaction perturbation. We perturbed the adenylate kinase reaction by increasing its V_{max} value by a factor of 5, and assessed the effect on the network, comparing metabolite levels, reaction fluxes, and insulin secretion to the unperturbed condition.

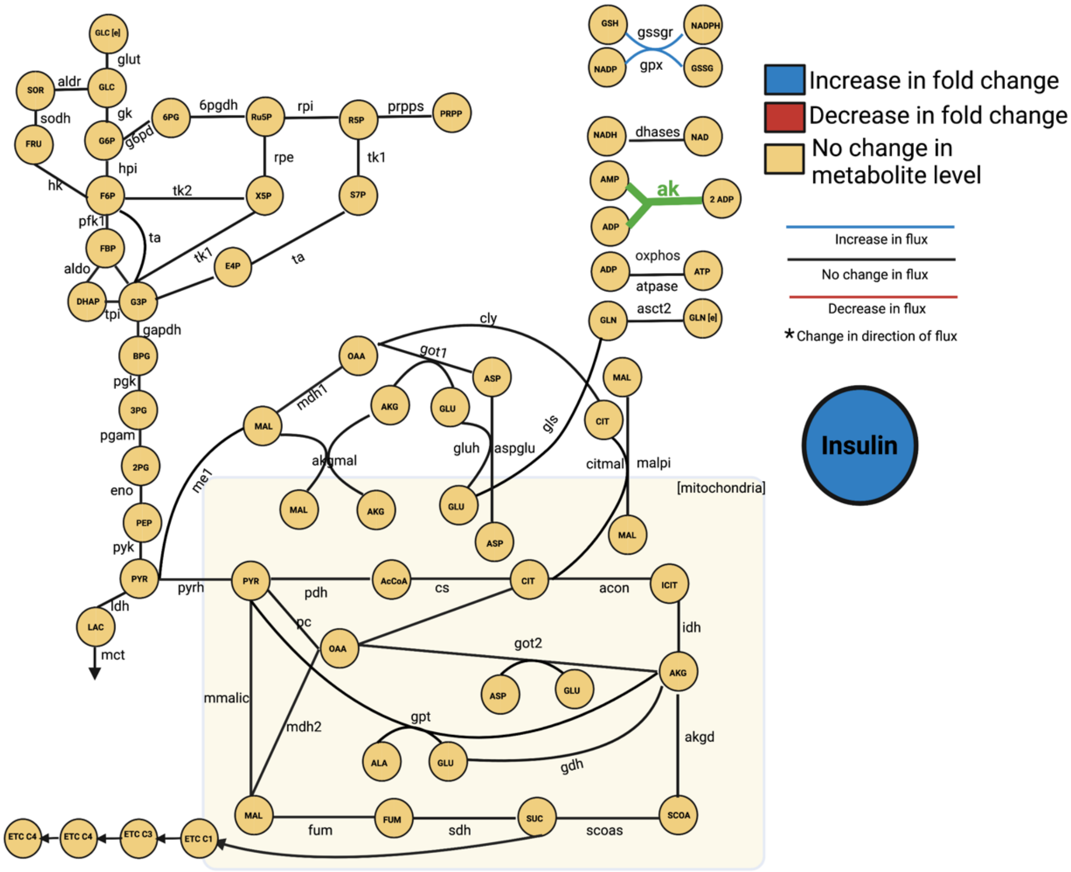

Supplement: S1 Supporting Information — (PDF) [file pcbi.1010555.s006.pdf]
