# Supplementary material for: Kinetic and data-driven modeling of pancreatic β-cell central carbon metabolism and insulin secretion
Source: PLoS Comput Biol. 2022 Oct 17;18(10):e1010555. doi: 10.1371/journal.pcbi.1010555 (PMC9612825; doi:10.1371/journal.pcbi.1010555)
Supplement: S1 Text — (DOCX) [file pcbi.1010555.s007.docx]

**S1 Text. Model equations**

**Model parameters**

Glycolysis pathway

Vf_glut = params(1,1);

Vf_gk = params(2,1);

Vf_hpi = params(3,1);

Vr_hpi = params(4,1);

Vf_pfk1 = params(5,1);

Vf_aldo = params(6,1);

Vr_aldo = params(7,1);

Vf_tpi = params(8,1);

Vf_gapdh = params(9,1);

Vf_pgk = params(10,1);

Vf_pgam = params(11,1);

Vf_eno = params(12,1);

Vf_pyk = params(13,1);

Vf_ldh = params(14,1);

Vr_ldh = params(15,1);

Vf_ak = params(16,1);

Vf_atpase = params(17,1);

Vr_atpase = params(18,1);

Vf_ox = params(19,1);

Vf_mct1 = params(20,1);

**PPP pathway**

Vf_g6pd = params(21,1);

Vr_g6pd = params(22,1);

Vf_6pgdh = params(23,1);

Vr_6pgdh = params(24,1);

Vf_rpe = params(25,1);

Vf_rpi = params(26,1);

Vf_prpps = params(27,1);

Vf_tk1 = params(28,1);

Vr_tk1 = params(29,1);

Vf_tk2 = params(30,1);

Vf_ta = params(31,1);

Vf_gpx = params(32,1);

Vf_gssgr = params(33,1);

Vr_gssgr = params(34,1);

**TCA cycle**

Vf_pdh = params(35,1);

Vf_cs = params(36,1);

Vf_acon = params(37,1);

Vf_idh = params(38,1);

Vf_akgd = params(39,1);

Vf_s = params(40,1);

Vf_sdh = params(41,1);

Vf_fum = params(42,1);

Vf_mdh2 = params(43,1);

Vf_got2 = params(44,1);

Vf_mdh1 = params(45,1);

Vf_got1 = params(46,1);

Vf_akgmal = params(47,1);

Vf_aspglu = params(48,1);

Vf_pyrh = params(49,1);

Vf_citmal = params(50,1);

Vf_malpi = params(51,1);

Vf_gluh = params(52,1);

Vf_cly = params(53,1);

Vf_malic = params(54,1);

Vf_cmalic = params(55,1);

Vf_pc = params(56,1);

Vf_gls = params(57,1);

Vf_gdh = params(58,1);

Vf_gpt = params(59,1);

Vf_asct2 = params(60,1);

f1_asct2 = params(61,1);

Kgluout_asct2 = params(62,1);

Kgluin_asct2 = params(63,1);

f2_asct2 = params(64,1);

Keq1_asct2 = params(65,1);

Kgluout1_asct2 = params(66,1);

Kgluin1_asct2 = params(67,1);

Vf_aconitase = params(68,1);

Vf_cIDH = params(69,1);

Vf_isocitmal = params(70,1);

Glu_out = params(71,1);

gk_K1GLC = params(72,1);

gk_K1ATP = params(73,1);

**Polyol Pathway**

Vf_aldr = params(74,1);

Vm_SoDH = params(75,1);

kfruT = params(76,1);

Ct_PyP = params(77,1);

Ct_Pyr = params(78,1);

k1_aldr = params(79,1);

Vm_r_SoDH = params(80,1);

Vf_hk = params(81,1);

**Electron Transport Chain**

Vmax_C1 = params(82,1);

Vmax_C3 = params(83,1);

Vmax_C4 = params(84,1);

Vmax_C5 = params(85,1);

%% The Vq values (setting the Vr values to constants) Based on parameter identifiability results

Vq_tpi = Vf_tpi/28;

Vq_gapdh = Vf_gapdh/0.413;

Vq_pgk = Vf_pgk/2.5;

Vq_pgam = Vf_pgam/0.36;

Vq_eno = Vf_eno/0.38;

Vq_ak = (Vf_ak/6)/13.34;

Vq_rpi = Vf_rpi/181.67;

Vq_rpe = Vf_rpe/1111.17;

Vq_tk2 = ((Vf_tk2)/60)/((1.23e+18)/60);

Vq_ta = ((Vf_ta)/60)/((7.53e+19)/60);

Vq_gpt = (Vf_gpt/60)/(1.6725e-06);

%% Fixed Concentration Values

mATP = 0.1;

mADP = 0.1;

mAMP = 0.1;

mH = 10^(-7.5);

cH = 10^(-7.2);

Mg = 0.7;

MgATP = 2.69;

mNAD = 10.10;

mNADH = 0.72;

mPi = 4.0;

cPi = 4.0;

mCOQ = 26;

mQH2 = 28;

mCO2 = 1.63;

cCO2 = 1.63;

mCoASH = 0.272;

cCoASH = 0.272;

cAcCoA = 0.030000;

K = 0.7;

mNH3 = 0.5;

Vm = 1;

Vc = 3;

F26BP = 0.0033;

H2o = 4.0;

**GLUT2 Reaction: Glu_out ⇔Glu_in**

km_glut = 42;

keq_glut = 1;

GLUT_reaction = ((Vf_glut/km_glut)*(Glu_out-(Glu_in/keq_glut)))...

/ (1 + (Glu_out/km_glut) + (Glu_in/km_glut));

**Glucokinase Equation: Gluin + ATP ⇔G6P + ADP**

GK_reaction = (Vf_gk*ATP*Glu_in/((gk_K1GLC+Glu_in)*(gk_K1ATP+ATP)));

**The HPI reaction : G6P ⇔F6P ; E4P, FBP, 6PG**

Kg6p_hpi = 0.4;

Kf6p_hpi = 0.05;

Kery4p_hpi = 0.001;

K6pg_hpi = 0.015;

Kfbp_hpi = 0.06;

HPI_reaction = ((Vf_hpi)*G6P/Kg6p_hpi - (Vr_hpi)*F6P/Kf6p_hpi)/...

( 1 + G6P/Kg6p_hpi + F6P/Kf6p_hpi + E4P/Kery4p_hpi + FBP/Kfbp_hpi + sixPG/K6pg_hpi);

**The PFK1 reaction :ATP + F6P ⇔FBP + ADP;**

Kf6p_pfk1 = 1.1;

Kf26bp_pfk1 = 0.00099;

Kiatp_pfk1 = 1.1;

Kcit_pfk1 = 6.7;

alpha_pfk1 = .75; %.75

beta_pfk1 = 1.18; %1.18

Katp_pfk1 = 0.0292;

L_pfk1 = 6.; %6.6

Kadp_pfk1 = .005;

Keq_pfk1 = 247;

Kfbp_pfk1 = 5; % 5

N1_PFK = ((F6P*(1+(F26BP/(alpha_pfk1*Kf26bp_pfk1))))/(Kf6p_pfk1*(1 + (F26BP/Kf26bp_pfk1))));

N2_PFK = (1 + ((F6P*(1+(F26BP/(alpha_pfk1*Kf26bp_pfk1))))/(Kf6p_pfk1*(1+(F26BP/Kf26bp_pfk1)))))^3;

D1_PFK = (L_pfk1*((1+(cCIT/Kcit_pfk1))^4)*((1 + (ATP/Kiatp_pfk1))^4))/((1+(F26BP/Kf26bp_pfk1))^4);

D2_PFK = ((1+((F6P*(1+(F26BP/(alpha_pfk1*Kf26bp_pfk1))))/(Kf6p_pfk1*(1+(F26BP/Kf26bp_pfk1)))))^4);

PFK1_reaction = (Vf_pfk1)*(0.2 + 0.8/(1 - 23.68/50))*(((ATP/Katp_pfk1)/(1+(ATP/Katp_pfk1)))* ((1+(beta_pfk1*F26BP/(alpha_pfk1*Kf26bp_pfk1)))/(1+(F26BP/(alpha_pfk1*Kf26bp_pfk1))))* (((N1_PFK*N2_PFK)/(D1_PFK + D2_PFK))...

- (((ADP*FBP)/(Kadp_pfk1*Kfbp_pfk1*Keq_pfk1))/(ADP/Kadp_pfk1 + FBP/Kfbp_pfk1 + ((ADP*FBP)/(Kadp_pfk1*Kfbp_pfk1)) + 1))));

**The Aldolase (ALDO) reaction: FBP ⇔ DHAP + G3P**

Kdhap_aldo = 0.08;

Kfbp_aldo = 0.009;

Kg3p_aldo = 0.16;

ALDO_reaction = (((Vf_aldo)*(FBP/Kfbp_aldo))-((Vr_aldo)*((DHAP*G3P)/(Kdhap_aldo*Kg3p_aldo))))/(1+ FBP/Kfbp_aldo +DHAP/Kdhap_aldo + G3P/Kg3p_aldo +((DHAP*G3P)/(Kdhap_aldo*Kg3p_aldo)));

**The TPI reaction: DHAP ⇔ G3P**

Kdhap_tpi = 1.6;

Kg3p_tpi = 0.51;

TPI_reaction = ((Vf_tpi)*(DHAP/Kdhap_tpi) - (Vq_tpi)*(G3P/Kg3p_tpi))/(1 + DHAP/Kdhap_tpi + G3P/Kg3p_tpi);

**The GAPDH reaction: NAD + G3P + Pi ⇔ 13BPG + NADH**

Kg3p_gapdh = 0.19;

Knad_gapdh = 0.09;

Knadh_gapdh = 0.01;

Kpi_gapdh = 11;

Kbpg_gapdh = 0.022;

GAPDH_reaction = (((Vf_gapdh)*((NAD*G3P*cPi)/(Knad_gapdh*Kg3p_gapdh*Kpi_gapdh)))-((Vq_gapdh)*((thirteenBPG*NADH)/(Kbpg_gapdh*Knadh_gapdh))))/...

(1 + NAD/Knad_gapdh +((NAD*G3P)/(Knad_gapdh*Kg3p_gapdh)) + ((NAD*G3P*cPi)/(Knad_gapdh*Kg3p_gapdh*Kpi_gapdh)) + ((thirteenBPG*NADH)/(Kbpg_gapdh*Knadh_gapdh))+(NADH/Knadh_gapdh));

**The PGK reaction: 13BPG + ADP ⇔ 3PG + ATP**

Ka_pgk = 0.079;

Kp_pgk = 0.13;

Kb_pgk = 0.04;

Kq_pgk = 0.27;

alpha_pgk = 1;

beta_pgk = 1;

PGK_reaction = (((Vf_pgk)*((thirteenBPG*ADP)/(alpha_pgk*Ka_pgk*Kb_pgk)))-((Vq_pgk)*((threePG*ATP)/(beta_pgk*Kp_pgk*Kq_pgk))))/...

(1 + (thirteenBPG/Ka_pgk) + (ADP/Kb_pgk) + ((thirteenBPG*ADP)/(alpha_pgk*Ka_pgk*Kb_pgk)) + ((threePG*ATP)/(beta_pgk*Kp_pgk*Kq_pgk)) + (threePG/Kp_pgk) + (ATP/Kq_pgk));

**The PGAM reaction: 3PG ⇔ 2PG**

K3pg_pgam = 0.19;

K2pg_pgam = 0.12;

PGAM_reaction = ((Vf_pgam)*threePG/K3pg_pgam - (Vq_pgam)*twoPG/K2pg_pgam)/(1+ threePG/K3pg_pgam+ twoPG/K2pg_pgam);

**The ENO reaction: 2PG ⇔ PEP**

K2pg_eno = 0.038;

Kpep_eno = 0.06;

ENO_reaction = ((Vf_eno)* twoPG/K2pg_eno - (Vq_eno)*PEP/Kpep_eno)/(1 + twoPG/K2pg_eno + PEP/Kpep_eno);

**The PYK reaction: PEP + ADP ⇔ Pyr + ATP**

Kadp_pyk = 0.4;

Katp_pyk = 0.86;

Keq_pyk = 1.9517e+05;

Kpep_pyk = 0.05;

Kpyr_pyk = 10;

PYK_reaction = Vf_pyk*(((PEP*ADP)/(Kpep_pyk*Kadp_pyk)) - ((Pyr*ATP)/(Kpep_pyk*Kadp_pyk*Keq_pyk)))/((1 + PEP/Kpep_pyk + Pyr/Kpyr_pyk)*(1 + ADP/Kadp_pyk + ATP/Katp_pyk));

**The LDH reaction: NADH + Pyr ⇔ Lacin + NAD**

k7 = 0.3;

LDH_reaction = (Vf_ldh*Pyr) / (k7 + Pyr);

**The AK reaction: ATP + AMP ⇔ 2ADP**

Kmakf = 5;

Kmakr = 2;

Nr_ak = (Vf_ak*((ATP*AMP)/Kmakf)) - ((Vq_ak)*((ADP*ADP)/Kmakr));

Dr_ak = 1 + ((ATP*AMP)/Kmakf) + ((ADP*ADP)/Kmakr);

AK_reaction = Nr_ak/Dr_ak;

**The ATPases reaction : ATP ⇔ ADP + Pi**

Katpf = 3;

Katpr = 4.71e-09;

Nr_atpase = ((Vf_atpase)*(ATP/Katpf)) - ((Vr_atpase)*((ADP*cPi*cH)/Katpr));

Dr_atpase = 1 + ATP/Katpf + (ADP*cPi*cH)/Katpr;

ATPase_reaction = Nr_atpase/Dr_atpase;

**The OxPhos : ADP + Pi ⇔ ATP**

Kmpyrox = 0.001;

Kmo2iox = 0.005;

Kmadpox = 0.005;

OxPhos_reaction = (Vf_ox*((Pyr/Kmpyrox)/(1 + Pyr/Kmpyrox))*((O2i/Kmo2iox)/(1 + O2i/Kmo2iox))*((ADP/Kmadpox)/(1 + ADP/Kmadpox)));

**The DHAses reaction: NAD ⇔ NADH**

Knadh_dhases = 250;

Knad_dhases = 1;

DHAses_reaction = (Knadh_dhases*NADH-Knad_dhases*NAD);

**The MCT reaction: Lac_in ⇔ Lac_out**

Keq_mct1 = 1;

Klacin_mct1 = 8.5;

Klacout_mct1 = 0.5;

MCT_reaction = (Vf_mct1*(Lac_in-(Lac_out/Keq_mct1)))/(Klacin_mct1*(1+(Lac_out/Klacout_mct1))+Lac_in);

**THE PPP REACTIONS**

**The G6PD reaction: G6P ⇔ 6PG**

G6PD_reaction = (((Vf_g6pd)*NADP*G6P - (Vr_g6pd)*sixPG*NADPH)/((1.45e+15) + (2.04e+20)*NADPH + (1.83e+20)*NADP + G6P*((4.29e+19) + (6.01e+24)*NADPH + (6.84e+24)*NADP) + sixPG*((5.74e+17) + (5.01e+24)*NADPH + (7.26e+22)*NADP + G6P*((1.10e+29)*NADPH + (8.65e+27)*NADP))));

**The 6PGDH reaction: 6PG ⇔ Ru5P**

sixPGDH_reaction = ((Vf_6pgdh)*NADP*sixPG - (Vr_6pgdh)*Ru5P*NADPH*cCO2)/...

((5.38e+15) + (7.86e+19)*NADPH + (6.97e+18)*NADP + Ru5P*((1.04e+18) + (3.99e+23)*NADPH + (3.03e+21)*NADP) + sixPG*((7.26e+18) + (2.40e+23)*NADPH + (4.46e+23)*NADP + Ru5P*((2.63e+27)*NADPH + (3.19e+27)*NADP)));

**RPE reaction: Ru5P ⇔ Xyl5P**

Kf_rpe = 0.19;

Kr_rpe = 0.5;

RPE_reaction = ((Vf_rpe)*(Ru5P/Kf_rpe) - (Vq_rpe)*(Xyl5P/Kr_rpe))/(1 + (Ru5P/Kf_rpe) + (Xyl5P/Kr_rpe));

**RPI reaction: Ru5P ⇔R5P**

Kf_rpi = 0.78;

Kr_rpi = 2.2;

RPI_reaction = ((Vf_rpi)*(Ru5P/Kf_rpi) - (Vq_rpi)*(R5P/Kr_rpi))/(1 + (Ru5P/Kf_rpi) + (R5P/Kr_rpi));

**PRPPS reaction R5P -> PRPP(null)**

Kmgatp_prpps = 0.01;

Kr5p_prpps = 0.57;

PRPPS_reaction = Vf_prpps*((MgATP*R5P)/((Kmgatp_prpps + MgATP)*(Kr5p_prpps + R5P)));

**TK1 reaction: R5P + Xyl5P ⇔G3P + S7P**

TK1_reaction = ((Vf_tk1)*Xyl5P*R5P - (Vr_tk1)*S7P*G3P)/((2.63e+16)*S7P + R5P*((4.40e+16) + (4.92e+16)*S7P) + (5.96e+16)*G3P + (6.94e+16)*S7P*G3P + Xyl5P*((7.35e+16) + (2.44e+17)*R5P + (3.38e+17)*G3P));

**TK2 reaction: E4P + Xyl5P ⇔F6P + G3P**

TK2_reaction = ((Vf_tk2)*Xyl5P*E4P - (Vq_tk2)*F6P*G3P)/(((3.10e+17)*E4P + (5.96e+16)*G3P +F6P*((1.25e+16) + (1.60e+17)*E4P + (3.31e+16)*G3P)) + Xyl5P*((7.35e+16) + (1.67e+18)*E4P + (3.38e+17)*G3P));

**TA reaction: S7P + G3P ⇔E4P + F6P**

TA_reaction = ((Vf_ta)*S7P*G3P - (Vq_ta)*F6P*E4P)/((3.4e+16)*S7P + (2.38e+16)*G3P + (1.64e+17)*E4P + (2.72e+15)*F6P + (4.41e+17)*S7P*G3P + (7.83e+16)*E4P*F6P + (2.21e+16)*F6P*G3P + (2.1e+18)*S7P*E4P);

**GPX reaction: GSH -> GSSG**

GPX_reaction = Vf_gpx*GSH;

**GSSGR reaction: GSSG ⇔GSH**

GSSGR_reaction = ((Vf_gssgr)*NADPH*GSSG - (Vr_gssgr)*NADP*GSH)/((1.73e+40) + (2.88e+42)*NADPH + (3.43e+41)*GSSG + (7.77e+35)*GSH + (2.47e+41)*NADP + (4.02e+43)*NADPH*GSSG + (1.3e+38)*NADPH*GSH + (4.9e+42)*NADP*GSSG + (5.55e+36)*GSH*GSH + (1.11e+37)*NADP*GSH + (1.24e+40)*NADP*GSH + (3.26e+39)*NADPH*GSSG*GSH + (2.08e+41)*NADPH*GSSG*GSH + (9.25e+38)*NADPH*GSH*GSH + (2.45e+41)*NADP*GSSG*GSH + (1.78e+39)*NADP*GSH*GSH + (2.32e+40)*NADPH*GSSG*GSH*GSH + (2.74e+40)*NADP*GSSG*GSH*GSH);

**The TCA cycle constants**

Khcit = 2.34e-03;

Kmgcit = 0.427;

Kkcit = 458;

Khatp = 2.57e-04;

Kmgatp = 0.151;

Kkatp = 13.5;

Khadp = 3.8e-04;

Kmgadp = 1.62;

Kkadp = 29.5;

Khamp = 6.03e-04;

Kmgamp = 13.8;

Kkamp = 89.1;

Khcoash = 7.41e-06;

Khscoa = 0.11;

Pcit = 1 + mH/Khcit + Mg/Kmgcit + K/Kkcit;

Patp = 1 + mH/Khatp + Mg/Kmgatp + K/Kkatp;

Padp = 1 + mH/Khadp + Mg/Kmgadp + K/Kkadp;

Pamp = 1 + mH/Khamp + Mg/Kmgamp + K/Kkamp;

Pfatp = 1 + mH/Khatp + K/Kkatp;

Pfadp = 1 + mH/Khadp + K/Kkadp;

Pfamp = 1 + mH/Khamp + K/Kkamp;

Pgtp = Patp;

Pfgtp = Pfatp;

Pgdp = Padp;

Pfgdp = Pfadp;

Pcoash = 1 + mH/Khcoash;

Pscoa = 1 + mH/Khscoa;

**Pyruvate Dehydrogenase : PDH reaction : mPyr ⇔mAcCoA**

Keq_pdh = 12000;

Kpyr_pdh = 0.0388;

Kcoash_pdh = 0.0099;

Knad_pdh = 0.0607;

Kiaccoa_pdh = 0.040;

Kinadh_pdh = 0.0402;

PDH_reaction = Vf_pdh*(mPyr*mCoASH*mNAD - (mAcCoA*mNADH*mCO2/Keq_pdh))/((Knad_pdh*(1 + mNADH/Kinadh_pdh)*mPyr*mCoASH) + (Kcoash_pdh*(1 + mAcCoA/Kiaccoa_pdh)*mPyr*mNAD) + Kpyr_pdh*mCoASH*mNAD + mPyr*mCoASH*mNAD);

**Citrate Synthase : CS_reaction: mAcCoA ⇔mCIT**

ka_cs = 5;

kb_cs = 4.5;

kia_cs = 5;

CS_reaction = (Vf_cs * mAcCoA * mOAA) / ((mAcCoA*OAA) + (ka_cs*mOAA) + (kb_cs*mAcCoA) + (kia_cs*kb_cs));

**Aconitase : ACON_reaction : mCIT ⇔mICIT**

Keq_acon = 0.027;

Kcit_acon = 1.161;

Kicit_acon = 0.434;

ACON_reaction = Vf_acon*(mCIT - mICIT/Keq_acon)/(Kcit_acon + mCIT + (Kcit_acon*mICIT)/Kicit_acon);

**Isocitrate Dehydrogenase : IDH : mICIT ⇔mAKG**

Keq_idh = 30.5;

Kmnad_idh = 0.074;

Kmicit_idh = 0.183;

Kaadp_idh = 0.050;

Kiatp_idh = 0.091;

Kiicit_idh = 0.0238;

Kinadh_idh = 0.029;

alpha_idh = (1 + (Kaadp_idh*Padp/(mADP*Pfadp))*(1 + mATP*Pfatp/(Kiatp_idh*Patp)));

IDH_reaction = (Vf_idh*(1 - ((mAKG*mNADH*mCO2)/(Keq_idh*mNAD*mICIT))))/...

(1 + (((Kmicit_idh/mICIT)^3)*alpha_idh) + ((Kmnad_idh/mNAD)*(1 + (((Kiicit_idh/mICIT)^3)*alpha_idh) + (mNADH/Kinadh_idh)*alpha_idh)));

**OXO-Glutarate dehydrogenase: AKGD : mAKG ⇔mSCOA**

Keq_akgd = 1.66e+08;

Kmakg_akgd = 0.120;

Kmcoash_akgd = 0.055;

Kmnad_akgd = 0.021;

Kaadp_akgd = 0.100;

Kiatp_akgd = 0.050;

Kiscoa_akgd = 0.0069;

Kinadh_akgd = 0.60e-03;

alpha_akgd = 1 + (Kaadp_akgd*Padp/(mADP*Pfadp))*(1 + ((mATP*Pfatp)/(Kiatp_akgd*Patp)));

AKGD_reaction = (Vf_akgd*(1 - ((mSCoA*mNADH*mCO2)/(Keq_akgd*mAKG*mNAD*mCoASH))))/...

(1 + ((Kmakg_akgd/mAKG)*alpha_akgd) + (Kmcoash_akgd/mCoASH)*(1 + (mSCoA/Kiscoa_akgd)) + ((Kmnad_akgd/mNAD)*(1 + (mNADH/Kinadh_akgd))));

**The SCOAS reaction: Succinyl CoA Synthetase: mSCOA + GDP ⇔mSUC + GTP**

Keq_s = 7.43;

Kmgdp_s = 0.016;

Kmscoa_s = 0.055;

Kmpi_s = 0.660;

Kmcoash_s = 0.020;

Kmsuc_s = 0.88;

Kmgtp_s = 0.0111;

Kigdp_s = 0.0055;

Kiscoa_s = 0.10;

Kipi_s = 0.2;

Kicoash_s = 0.020;

Kisuc_s = 0.3;

Kigtp_s = 0.0111;

N_scoas = Vf_s*(mGDP*mSCoA*mPi - (mCoASH*mSUC*mGTP)/Keq_s);

D1_scoas = Kigdp_s*Kiscoa_s*Kmpi_s;

D2_scoas = Kiscoa_s*Kmpi_s*mGDP;

D3_scoas = Kigdp_s*Kmscoa_s*mPi;

D4_scoas = Kmpi_s*mGDP*mSCoA;

D5_scoas = Kmscoa_s*mGDP*mPi;

D6_scoas = Kmgdp_s*mSCoA*mPi;

D7_scoas = mSCoA*mGDP*mPi;

D8_scoas = (Kigdp_s*Kiscoa_s*Kmpi_s)/(Kisuc_s*Kigtp_s*Kmcoash_s);

D9_scoas = Kigtp_s*Kmsuc_s*mCoASH;

D10_scoas = Kisuc_s*Kmcoash_s*mGTP;

D11_scoas = Kmgtp_s*mCoASH*mSUC;

D12_scoas = Kmsuc_s*mCoASH*mGTP;

D13_scoas = Kmcoash_s*mSUC*mGTP;

D14_scoas = mCoASH*mSUC*mGTP;

D15_scoas = (Kmsuc_s*Kigtp_s*mGDP*mSCoA*mSUC)/(Kigdp_s*Kiscoa_s);

D16_scoas = (Kmgtp_s*mCoASH*mGDP*mSUC)/(Kigdp_s);

D17_scoas = (Kigtp_s*Kmsuc_s*mGDP*mSCoA*mPi*mCoASH)/(Kigdp_s*Kiscoa_s*Kipi_s);

D18_scoas = (Kmsuc_s*Kigtp_s*mGDP*mCoASH)/(Kigdp_s);

D19_scoas = (Kicoash_s*Kmgtp_s*mGDP*mSCoA*mPi*mSUC)/(Kigdp_s*Kiscoa_s*Kipi_s);

D20_scoas = (Kmgtp_s*mGDP*mSCoA*mSUC*mCoASH)/(Kigdp_s*Kiscoa_s);

D21_scoas = (Kmgtp_s*mGDP*mSCoA*mPi*mCoASH*mSUC)/(Kigdp_s*Kiscoa_s*Kipi_s);

D22_scoas = (Kigdp_s*Kmscoa_s*mGTP*mPi*mSUC)/(Kisuc_s*Kigtp_s);

D23_scoas = (Kmgdp_s*Kipi_s*mGTP*mSCoA*mSUC*mCoASH)/(Kicoash_s*Kisuc_s*Kigtp_s);

D24_scoas = (Kigdp_s*Kmscoa_s*mGTP*mSUC*mPi*mCoASH)/(Kicoash_s*Kisuc_s*Kigtp_s);

D25_scoas = (Kmgdp_s*Kmscoa_s*mGTP*mPi)/(Kigtp_s);

D26_scoas = (Kigdp_s*Kmscoa_s*mGTP*mPi)/(Kigtp_s);

D27_scoas = (Kmgdp_s*mSCoA*mSUC*mGTP*mPi)/(Kisuc_s*Kigtp_s);

D28_scoas = (Kmgdp_s*mSCoA*mSUC*mGTP*mPi*mCoASH)/(Kiscoa_s*Kigdp_s*Kipi_s);

D_scoas = D1_scoas + D2_scoas + D3_scoas + D4_scoas + D5_scoas + D6_scoas + D7_scoas

+ D8_scoas*(D9_scoas + D10_scoas + D11_scoas + D12_scoas + D13_scoas + D14_scoas + D15_scoas + D16_scoas + D17_scoas + D18_scoas + D19_scoas + D20_scoas + D21_scoas)...

+ D22_scoas + D23_scoas + D24_scoas + D25_scoas + D26_scoas + D27_scoas + D28_scoas;

SCOAS_reaction = N_scoas/D_scoas;

**Succinate dehydrogenase:SDH : mSUC ⇔ mFUM**

Kmsuc_sdh = 0.467;

Kmcoq_sdh = 0.480;

Kmqh2_sdh = 0.00245;

Kmfum_sdh = 1.200;

Kisuc_sdh = 0.120;

Kifum_sdh = 1.275;

Keq_sdh = 1.21;

Kioaa_sdh = 0.0015;

Kasuc_sdh = 0.45;

Kafum_sdh = 0.375;

alpha_sdh = (1 + mOAA/Kioaa_sdh + mSUC/Kasuc_sdh + mFUM/Kafum_sdh)/...

(1 + mSUC/Kasuc_sdh + mFUM/Kafum_sdh);

Nr_sdh = Vf_sdh*(mSUC*mCOQ - (mQH2*mFUM/Keq_sdh));

Dr_sdh = Kisuc_sdh*Kmcoq_sdh*alpha_sdh + Kmcoq_sdh*mSUC + Kmsuc_sdh*alpha_sdh*mCOQ ...

+ mSUC*mCOQ + (Kmsuc_sdh/Kifum_sdh)*mCOQ*mFUM ...

+ (Kisuc_sdh*Kmcoq_sdh/(Kifum_sdh*Kmqh2_sdh))*(Kmfum_sdh*alpha_sdh*mQH2 + Kmqh2_sdh*mFUM + (Kmfum_sdh/Kisuc_sdh)*mSUC*mQH2 + mQH2*mFUM);

SDH_reaction = Nr_sdh/Dr_sdh;

**Fumarate hydratase: FUM : mFUM ⇔mMAL**

Kmfum_fum = 0.0447;

Kmmal_fum = 0.1977;

Kicit_fum = 3.5;

Kiatp_fum = 0.040;

Kiadp_fum = 0.400;

Kigtp_fum = 0.080;

Kigdp_fum = 0.33;

Keq_fum = 4.56;

alpha_fum = (1 + mCIT/Kicit_fum + (mATP*Pfatp)/(Kiatp_fum*Patp) + (mADP*Pfadp)/(Kiadp_fum*Padp) + (mGTP*Pfgtp)/(Kigtp_fum*Pgtp) + (mGDP*Pfgdp)/(Kigdp_fum*Pgdp));

Nr_fum = Vf_fum*(mFUM - (mMal/Keq_fum));

Dr_fum = Kmfum_fum*alpha_fum + mFUM + (Kmfum_fum*mMal)/Kmmal_fum;

FUM_reaction = Nr_fum/Dr_fum;

**Malate Dehydrogenase 2: MDH2 : mMAL ⇔mOAA**

Keq_mdh2 = 4.02e-04;

Kmnad_mdh2 = 0.09055;

Kmmal_mdh2 = 0.250;

Kmoaa_mdh2 = 0.006128;

Kmnadh_mdh2 = 0.00258;

Kinad_mdh2 = 0.279;

Kimal_mdh2 = 0.360;

Kioaa_mdh2 = 0.0055;

Kinadh_mdh2 = 0.00318;

Kiatp_mdh2 = 0.1832;

Kiadp_mdh2 = 0.3944;

Kiamp_mdh2 = 0.420;

alpha_mdh2 = 1 + (mATP*Pfatp)/(Kiatp_mdh2*Patp) + (mADP*Pfadp)/(Kiadp_mdh2*Padp) + (mAMP*Pfamp)/(Kiamp_mdh2*Pamp);

MDH2_reaction = (Vf_mdh2*(mNAD*mMal - ((mOAA*mNADH)/Keq_mdh2)))/...

(Kinad_mdh2*Kmmal_mdh2*alpha_mdh2 + Kmmal_mdh2*mNAD + Kmnad_mdh2*mMal*alpha_mdh2 + mMal*mNAD + ((Kmnad_mdh2*mMal*mNADH)/Kinadh_mdh2) + ((mOAA*mNAD*mMal)/Kioaa_mdh2) + ((Kinad_mdh2*Kmmal_mdh2)/(Kinadh_mdh2*Kmoaa_mdh2))*(Kmnadh_mdh2*mOAA*alpha_mdh2 + Kmoaa_mdh2*mNADH + ((Kmnadh_mdh2*mOAA*mNAD)/Kinad_mdh2) + mNADH*mOAA + ((mMal*mOAA*mNADH)/Kimal_mdh2)));

**Glutamine Oxaloacetate Transaminase 2 (GOT2): mAKG + mASP ⇔mGLU + mOAA**

Keq_got2 = 1.56;

Kmasp_got2 = 0.89;

Kmakg_got2 = 3.22;

Kmoaa_got2 = 0.088;

Kmglu_got2 = 32.5;

Kiasp_got2 = 3.9;

Kiglu_got2 = 10.7;

KiAKG_got2 = 26.5;

GOT2_reaction = (Vf_got2*(mASP*mAKG - (mOAA*mGlutamate/Keq_got2)))/...

(Kmakg_got2*mASP + Kmasp_got2*(1 + mAKG/KiAKG_got2)*mAKG + mASP*mAKG + (Kmasp_got2*mAKG*mGlutamate/Kiglu_got2) + ((Kiasp_got2*Kmakg_got2)/(Kmoaa_got2*Kiglu_got2))*((Kmglu_got2*mASP*mOAA/Kiasp_got2) + mOAA*mGlutamate + Kmglu_got2*(1 + mAKG/KiAKG_got2)*mOAA + Kmoaa_got2*mGlutamate));

**Malate Dehydrogenase 1 (MDH1): OAA + NADH ⇔MAL + NAD**

Keq_mdh1 = 2.67e+04;

Kmnad_mdh1 = 0.114;

Kmmal_mdh1 = 1.1;

Kmoaa_mdh1 = 0.088;

Kmnadh_mdh1 = 0.026;

Kinadh_mdh1 = 0.0049;

Kioaa_mdh1 = 0.063;

Kimal_mdh1 = 7.1;

Kinad_mdh1 = 0.094;

MDH1_reaction = (Vf_mdh1*(NADH*OAA - (NAD*cMal)/Keq_mdh1))/ ...

(Kinadh_mdh1*Kmoaa_mdh1 + Kmoaa_mdh1*NADH + Kmnadh_mdh1*OAA + OAA*NADH + ((Kmnadh_mdh1*OAA*NAD)/Kinad_mdh1) + ((OAA*NADH*cMal)/Kimal_mdh1) + ((Kinadh_mdh1*Kmoaa_mdh1)/(Kinad_mdh1*Kmmal_mdh1))*(Kmnad_mdh1*cMal + Kmmal_mdh1*NAD + ((Kmnad_mdh1*cMal*NADH)/Kinadh_mdh1) + NAD*cMal + ((cMal*OAA*NAD)/Kioaa_mdh1)));

**Glutamate Oxaloactate Transaminase 1 (GOT1) : ASP + AKG ⇔OAA + GLU**

Keq_got1 = 1.56;

Kmasp_got1 = 4.4;

Kmakg_got1 = 0.38;

Kmoaa_got1 = 0.095;

Kmglu_got1 = 9.6;

Kiasp_got1 = 3.9;

Kiglu_got1 = 8.4;

KiAKG_got1 = 26.5;

GOT1_reaction = (Vf_got1*(ASP*AKG - (OAA*Glutamate/Keq_got1)))/...

(Kmakg_got1*ASP + Kmasp_got1*(1 + AKG/KiAKG_got1)*AKG + ASP*AKG + (Kmasp_got1*AKG*Glutamate/Kiglu_got1) + ((Kiasp_got1*Kmakg_got1)/(Kmoaa_got1*Kiglu_got1))*((Kmglu_got1*ASP*OAA/Kiasp_got1) + OAA*Glutamate + Kmglu_got1*(1 + AKG/KiAKG_got1)*OAA + Kmoaa_got1*Glutamate));

**Alpha-Ketoglutarate-Malate Transporter : AKGMAL : AKG + mMAL ⇔mAKG + cMAL**

Kmmali_akgmal = 0.4;

Kmmalx_akgmal = 10;

Kmakgi_akgmal = 1.3;

Kmakgx_akgmal = 0.17;

AKGMAL_reaction = (Vf_akgmal*(AKG*mMal - mAKG*cMal))/...

(Kmakgi_akgmal*Kmmalx_akgmal*(2 + cMal/Kmmali_akgmal + mMal/Kmmalx_akgmal + AKG/Kmakgi_akgmal + mAKG/Kmakgx_akgmal + (cMal*mAKG)/(Kmmali_akgmal*Kmakgx_akgmal) + (mMal*AKG)/(Kmmalx_akgmal*Kmakgi_akgmal)));

**Aspartate-Glutamate Transporter: ASPGLU : mGLU + ASP ⇔GLU + mASP**

Keq_aspglu = 0.6;

Kiaspi_aspglu = 0.028;

Kiaspx_aspglu = 2.8;

Kiglui_aspglu = 0.18;

Kiglux_aspglu = 1.6;

Kh_aspglu = 10^(-6.5);

M_aspglu = 1.8;

ASPGLU_reaction = (Vf_aspglu*(Keq_aspglu*ASP*mGlutamate*mH - mASP*Glutamate*cH))/...

(Keq_aspglu*Kiaspi_aspglu*Kiglux_aspglu*Kh_aspglu*(2*M_aspglu + M_aspglu*ASP/Kiaspi_aspglu + ((ASP*mGlutamate*mH)/(Kiaspi_aspglu*Kiglux_aspglu*Kh_aspglu)) + M_aspglu*((mASP*cH)/(Kiaspx_aspglu*Kh_aspglu)) + M_aspglu*mASP/Kiaspx_aspglu + ((mASP*Glutamate*cH)/(Kiaspx_aspglu*Kiglui_aspglu*Kh_aspglu)) + ((M_aspglu*ASP*mH)/(Kiaspi_aspglu*Kh_aspglu)) + M_aspglu*mH/Kh_aspglu + ((M_aspglu*Glutamate*cH)/(Kiglui_aspglu*Kh_aspglu)) + M_aspglu*cH/Kh_aspglu + ((M_aspglu*mGlutamate*mH)/(Kiglux_aspglu*Kh_aspglu))));

**The PYRH reaction: Pyruvate Hydrogen Shuttle: cPYR ⇔mPYR**

PYRH_reaction =(Vf_pyrh*(Pyr*cH - mPyr*mH));

**The CITMAL reaction: Citrate Malate Shuttle: cCIT + mMAL ⇔ mCIT + cMAL**

CITMAL_reaction =Vf_citmal*(cCIT*mMal - mCIT*cMal);

**The ISOCITMAL reaction : ICIT malate reaction : cICIT + mMAL ⇔ mICIT + cMAL**

alpha = 1;

KiS1 = 0.13;

KiS2 = 0.44;

KcF = 5.6;

beta = 1;

KiP1 = 0.33;

KiP2 = 4.18e-02;

KcR = 3.5;

gamma = 1;

delta = 1;

ISOCITMAL_reaction = Vf_isocitmal*((cICIT*mMal/alpha/KiS1/KiS2*KcF-cMal*mICIT/beta/KiP1/KiP2*KcR)/(1+cICIT/KiS1+mMal/KiS2+cMal/KiP1+mICIT/KiP2+cICIT*mMal/alpha/KiS1/KiS2+cMal*mICIT/beta/KiP1/KiP2+mMal*mICIT/gamma/KiS2/KiP2+cICIT*cMal/delta/KiS1/KiP1));

**Malate-Phosphate shuttle(MALPi reaction): cMAL -> mMAL**

MALPi_reaction = Vf_malpi*(cMal*mPi - mMal*cPi);

**The Glutamate-hydrogen shuttle(GLUH reaction): cGLU -> mGLU**

GLUH_reaction = Vf_gluh*(Glutamate*cH - mGlutamate*mH);

**ATP-Citrate Lyase: CLY : cCIT -> cOAA**

Kmcit_cly = 0.0493;

Kicit_cly = 0.0475;

Kmcoash_cly = 0.0044;

Kicoash_cly = 0.0061;

Kmoaa_cly = 0.177;

Kioaa_cly = 0.177;

Kmaccoa_cly = 0.0098;

Kiaccoa_cly = 0.0098;

CLY_reaction = (Vf_cly*cCIT*cCoASH)/...

(Kicit_cly*Kmcoash_cly + Kmcit_cly*cCoASH ...

+ Kmaccoa_cly*cCIT + ...

cCIT*cCoASH + ...

(Kmcit_cly*cCoASH*cAcCoA)/Kiaccoa_cly ...

+ (cCIT*cCoASH*OAA)/Kioaa_cly + ...

(Kicit_cly*Kmcoash_cly/(Kmoaa_cly*Kiaccoa_cly))*...

(Kmaccoa_cly*OAA + Kmoaa_cly*cAcCoA + (Kmaccoa_cly*cCIT*OAA)/Kicit_cly + OAA*cAcCoA + (cCoASH*OAA*cAcCoA)/Kicoash_cly));

**Malic Enzyme 2 (Mitochondrial): mMAL ⇔mPYR**

Keq_malic = 34.4;

Kmal_malic = 1.7;

Knad_malic = 0.16;

Kiatp_malic = 0.5;

MMALIC_reaction = Vf_malic*(mMal*mNAD - (mPyr*mNADH*mCO2/Keq_malic))/...

(Kmal_malic*mNAD*(1 + mATP/Kiatp_malic) + Knad_malic*mMal + Kmal_malic*Knad_malic);

**Malic Enzyme 1 (Cytosolic):cMAL +NADP ⇔PYR + NADPH**

Kmmal_cmalic = 0.120;

Kmnadp_cmalic = 0.0014;

Kmco2_cmalic = 0.013;

Kmpyr_cmalic = 0.0064;

Kmnadph_cmalic = 0.0021;

Kinadp_cmalic = 0.00096;

Kimal_cmalic = 0.22;

Kico2_cmalic = 0.0117;

Kipyr_cmalic = 7.8;

Kinadph_cmalic = 0.002;

Keq_cmalic = 34.4;

Nr_cmalic = Vf_cmalic*(cMal*NADP - (cCO2*Pyr*NADPH)/Keq_cmalic);

D1 = Kinadp_cmalic*Kmmal_cmalic;

D2 = Kmmal_cmalic*NADP;

D3 = Kmnadp_cmalic*cMal;

D4 = cMal*NADP;

D5 = (Kinadp_cmalic*Kmmal_cmalic*Kmpyr_cmalic*cCO2)/(Kmco2_cmalic*Kipyr_cmalic);

D6 = (Kinadp_cmalic*Kmmal_cmalic*NADPH)/(Kinadph_cmalic);

D7 = (Kmmal_cmalic*Kmnadph_cmalic*NADP*Pyr)/(Kipyr_cmalic*Kinadph_cmalic);

D8 = cCO2/Kmco2_cmalic + (Kico2_cmalic*cMal/Kimal_cmalic*Kmco2_cmalic) + (cMal*cCO2/Kimal_cmalic*Kmco2_cmalic);

D9 = (Kmnadp_cmalic*cMal*NADPH/Kinadph_cmalic)*(1 + Pyr/Kipyr_cmalic + (cCO2*Pyr)/(Kico2_cmalic*Kipyr_cmalic));

D10 = ((Kmmal_cmalic*Kinadp_cmalic*Kmpyr_cmalic*NADPH)/(Kipyr_cmalic*Kinadph_cmalic))*(Pyr/Kmpyr_cmalic + cCO2/Kmco2_cmalic);

D11 = ((Kmmal_cmalic*Kinadp_cmalic*Kmnadph_cmalic*cCO2*Pyr)/(Kmco2_cmalic*Kipyr_cmalic*Kinadph_cmalic))*(1 + NADPH/Kmnadph_cmalic);

D12 = ((Kmmal_cmalic*Kmpyr_cmalic*NADP*cCO2)/(Kmco2_cmalic*Kipyr_cmalic))*(1 + cMal/Kimal_cmalic);

CMALIC_reaction = Nr_cmalic/(D1 + D2 + D3 + D4 + D5 + D6 + D7*D8 + D9 + D10 + D11 + D12);

**Pyruvate carboxylase reaction: Pyruvate to Oxaloacetate: mPYR ⇔mOAA**

Keq_pc = 1;

Kmpyr_pc = 0.22;

Kmco2_pc = 3.2;

PC_reaction = Vf_pc*(mPyr*mCO2 - mOAA/Keq_pc)/(Kmpyr_pc*Kmco2_pc + Kmpyr_pc*mCO2 + Kmco2_pc*mPyr + mPyr*mCO2);

**Glutaminase : GLS : Gln ⇔ mGlu**

Keq_gls = 1;

Kmgln_gls = 12;

Kiglu_gls = 55;

GLS_reaction =(Vf_gls*(Glutamine_in - (mGlutamate/Keq_gls)))/...

(Kmgln_gls*(1 + (mGlutamate/Kiglu_gls)) + Glutamine_in);

**Glutamate Dehydrogenase: GDH : mGlu ⇔ mAKG**

Kmglu_gdh = 3.5;

Kmnadh_gdh = 0.04;

Kmakg_gdh = 1.1;

Kmnh3_gdh = 6;

Kiakg_gdh = 0.25;

Kinh3_gdh = 6;

Kiglu_gdh = 3.5;

Kinadh_gdh = 0.004;

Kinad_gdh = 1;

Keq_gdh = 0.003;

Kmnad_gdh = 1;

N_gdh = Vf_gdh*(mNAD*mGlutamate - (mAKG*mNADH*mNH3)/Keq_gdh);

D1_gdh = Kinad_gdh*Kmglu_gdh;

D2_gdh = Kmglu_gdh*mNAD;

D3_gdh = Kmnad_gdh*mGlutamate;

D4_gdh = mGlutamate*mNAD;

D5_gdh = (mGlutamate*mNAD*mNH3)/Kiakg_gdh;

D6_gdh = (Kinad_gdh*Kmglu_gdh*mNADH)/Kinadh_gdh;

D7_gdh = (Kmglu_gdh*mNH3*mNAD)/Kinh3_gdh;

D8_gdh = (Kinad_gdh*Kmglu_gdh*Kmnadh_gdh*mNH3*mAKG)/(Kmnh3_gdh*Kinadh_gdh*Kiakg_gdh);

D9_gdh = (Kmnad_gdh*mGlutamate*mNADH)/Kinadh_gdh;

D10_gdh = (mGlutamate*mNAD*mNH3*mAKG)/(Kinh3_gdh*Kiakg_gdh);

D11_gdh = (Kinad_gdh*Kmglu_gdh*Kmakg_gdh*mNH3*mNADH)/(Kmnh3_gdh*Kinadh_gdh*Kiakg_gdh);

D12_gdh = (Kinad_gdh*Kmglu_gdh*Kmakg_gdh*mNH3*mNADH*mAKG)/(Kmnh3_gdh*Kinadh_gdh*Kiakg_gdh);

D13_gdh = (mGlutamate*mNAD*mAKG)/Kiakg_gdh;

D14_gdh = (Kinad_gdh*Kmglu_gdh*Kmakg_gdh*mNH3)/(Kmnh3_gdh*Kiakg_gdh);

D15_gdh = (Kinad_gdh*Kmglu_gdh*mGlutamate*mNADH*mAKG)/(Kiglu_gdh*Kinadh_gdh*Kiakg_gdh);

D16_gdh = (Kinad_gdh*Kmglu_gdh*mNADH*mAKG)/(Kinadh_gdh*Kiakg_gdh);

D17_gdh = (Kmnadh_gdh*Kmglu_gdh*mNH3*mNAD*mAKG)/(Kmnh3_gdh*Kinadh_gdh*Kiakg_gdh);

D18_gdh = (Kinad_gdh*Kmglu_gdh*mGlutamate*mNH3*mNADH*mAKG)/(Kmnh3_gdh*Kiglu_gdh*Kinadh_gdh*Kiakg_gdh);

D_gdh = D1_gdh + D2_gdh + D3_gdh + D4_gdh + D5_gdh + D6_gdh + D7_gdh ...

+ D8_gdh + D9_gdh + D10_gdh + D11_gdh + D12_gdh + D13_gdh + D14_gdh + D15_gdh + D16_gdh + D17_gdh + D18_gdh;

GDH_reaction = N_gdh/D_gdh;

**GPT reaction: Glutamine Pyruvate transaminase: mAKG + mALA ⇔ mPyr + mGlutamate**

Kala_gpt = 3.0;

Kakg_gpt = 0.12;

Kpyr_gpt = 0.23;

Kglu_gpt = 8.1;

Kipyr_gpt = 0.23;

Kiglu_gpt = 2.8;

Kia_gpt = 470;

Kig_gpt = 96;

Krg_gpt = 79.16;

Keq_gpt = 2.2;

GPT_reaction = (Vf_gpt*(mALA*mAKG - (mPyr*mGlutamate)/Keq_gpt))/...

(Kala_gpt*mAKG + Kakg_gpt*mALA + mAKG*mALA + ...

Kala_gpt*mAKG*mGlutamate/Kiglu_gpt + ...

Kakg_gpt*mALA*mALA/Kia_gpt + ...

Kakg_gpt*mALA*mGlutamate/Krg_gpt + ...

(Vf_gpt/((Vf_gpt/Vq_gpt)*Keq_gpt))*(Kpyr_gpt*mGlutamate + Kglu_gpt*mPyr + mPyr*mGlutamate + Kakg_gpt*mALA*mPyr/Kipyr_gpt + Kpyr_gpt*mGlutamate*mGlutamate/Kig_gpt));

**The ASCT2 reaction: Gln_out ⇒ Gln_in**

Kmax_asct2 = 0.097;

ASCT2_reaction = (Vf_asct2)*((f1_asct2*(Glutamine_out-(Glutamine_in/Kmax_asct2)))/(Kgluout_asct2*(1 + (Glutamine_in/Kgluin_asct2)) + Glutamine_out) + ...

(f2_asct2*(Glutamine_out - (Glutamine_in/Keq1_asct2)))/(Kgluout1_asct2*(1 + (Glutamine_in/Kgluin1_asct2)) + Glutamine_out));

**Aconitase2 reaction : cCIT ⇔ cICIT**

KcF = 20.47;

Kp = 0.00011;

KcR = 31.44;

Ks = 0.0005;

Aconitase2_reaction = Vf_aconitase*(KcF*Kp*cCIT-KcR*Ks*cICIT)/(Ks*cICIT+Kp*cCIT+Ks*Kp);

**Cytosolic isocitrate dehydrogenase : cICIT + NADP ⇔ cAKG + NADPH**

phi0 = 0.051;

phi1 = 9.5e-08;

phi2 = 9.6e-07;

phi12 = 9e-08;

phir0 = 0.066;

phir1 = 3.7e-07;

phir2 = 2.9e-05;

phir3 = 0.00025;

phir12 = 6e-12;

phir13 = 1.3e-10;

phir23 = 9.4e-08;

phir123 = 4.6e-14;

cIDH_reaction = Vf_cIDH*(cICIT*NADP/(phi0*cICIT*NADP+phi1*NADP+phi2*cICIT+phi12)-AKG*NADPH*cCO2/(phir0*AKG*NADPH*cCO2+phir1*NADPH*cCO2+phir2*AKG*cCO2+phir3*AKG*NADPH+phir12*cCO2+phir13*NADPH+phir23*AKG+phir123));

**The Oxtransfer : O2e ⇔O2i**

ko2 = 2.74;

Oxtransfer_reaction = ko2*(O2e-O2i);

**Aldose Reductase: Glu_in ⇔ Sor**

k2_aldr = 0.037;

ka_aldr = 6.9E-4;

kb_aldr = 46;

kp_aldr = 3.8E2;

kq_aldr = 1.5E-2;

kia_aldr = 4.1E-4;

kib_aldr = 9.2E-2;

kip_aldr = 1.3E3;

kiq_aldr = 8.3E-3;

Keq_aldr = (k1_aldr*kp_aldr*kiq_aldr)/(k2_aldr*kb_aldr*kia_aldr);

ALDR_reaction = Vf_aldr * (k1_aldr*NADPH*Glu_in - (k2_aldr*(Ct_PyP - NADPH)*Sor)/Keq_aldr) ...

/ ((k2_aldr*kia_aldr*kb_aldr + k2_aldr*kb_aldr*NADPH + k2_aldr*ka_aldr*Glu_in + ...

(k1_aldr*kq_aldr/Keq_aldr)*Sor + (k1_aldr*kp_aldr/Keq_aldr)*(Ct_PyP - NADPH) + ...

(k2_aldr*Glu_in*NADPH) + (k1_aldr*kq_aldr/(kia_aldr*Keq_aldr))*Sor*(Ct_PyP-NADPH) + ...

(k2_aldr*ka_aldr/kiq_aldr)*Glu_in*(Ct_PyP - NADPH) + (k1_aldr/Keq_aldr)*Sor*NADPH + ...

(k2_aldr/kip_aldr)*Glu_in*NADPH*Sor + (k1_aldr/(kib_aldr*Keq_aldr))*Glu_in*(Ct_PyP-NADPH)*Sor));

**Sorbitol Dehydrogenase (SoDH) Reaction: Sor ⇔ Fru**

Km_NadhSodh = 1E-2;

Km_FruSodh = 1E3;

Km_SorSodh = 9.6;

Km_NadSodh = 2.0;

Ki_NadhSodh = 1E-2;

Ki_NadSodh = 2.0;

Keq_Sodh = 9.25E-2;

SoDH_reaction = Vm_SoDH*((Ct_Pyr-NADH)*Sor / (Ki_NadSodh*Km_SorSodh+Km_SorSodh*(Ct_Pyr-NADH) + Km_NadSodh*Sor + ...

(Ct_Pyr - NADH)*Sor)) - Vm_r_SoDH*((NADH*Fru)/(Ki_NadhSodh*Km_FruSodh+ Km_FruSodh*NADH + Km_NadhSodh*Fru+ ...

NADH*Fru));

**Fructose Transport: Fru ⇒Fru_e (null)**

KfrufruT = 3.0;

VfruT = (kfruT *Fru)/(KfrufruT + Fru);

**Respiratory Chain/ETC**

**Complex 1: NADH + Q + 5H ⇔ NAD + QH_2_ + 4H**

Kcf_C1 = 498;

Kcr_C1 = 229;

Keq_C1 = 407.9;

Kms1_C1 = 9.2E-6;

Kms2_C1 = 2.6E-4;

Kmp1_C1 = 9.9E-6;

Kmp2_C1 = 5.9E-5;

KiS1_C1 = 2.1E-8;

KiP2_C1 = 9.8E-8;

denom1 = (Kcr_C1*Kms2_C1*NADH) + (Kcr_C1*Kms1_C1*mCOQ) + ((Kcf_C1*Kmp2_C1*NAD)/Keq_C1) + ((Kcf_C1*Kmp1_C1*mQH2)/Keq_C1) + (Kcr_C1*NADH*mCOQ) + ((Kcf_C1*Kmp2_C1*NADH*NAD)/(Keq_C1*KiS1_C1)) + ((Kcf_C1*NAD*mQH2) / Keq_C1) + ((Kcr_C1*Kms1_C1*mCOQ*mQH2)/KiP2_C1);

Complex1_reaction = (Vmax_C1*Kcf_C1*Kcr_C1*(NADH*mCOQ - ((NAD*mQH2)/Keq_C1))) / denom1;

**Complex 3: QH_2_ + 2cyt_c3 + 2H ⇔ Q + 2cyt_c2 + 4H**

Kcf_C3 = 426.8;

Kma_C3 = 2.8E-5;

Kmb_C3 = 3.0E-6;

Kb1_C3 = 5.4E-6;

Kb2_C3 = 5.7E-6;

Kq1_C3 = 2.8E-6;

Kq2_C3 = 1.9E-6;

K8_C3 = 622.1;

denom3 = (mCOQ*((Kma_C3*Kq2_C3*Kb2_C3) + (Kma_C3*Kq2_C3*Cyt_c3) + ((Kcf_C3/K8_C3)*Kq1_C3*mQH2*Kb1_C3) + ((Kcf_C3/K8_C3)*Kq1_C3*mQH2*Cyt_c3)))...

+ (Kma_C3*Cyt_c3) + (Kmb_C3*mQH2) + (mQH2*Cyt_c3);

Complex3_reaction = (Vmax_C3*Kcf_C3*mQH2*Cyt_c3) / denom3;

**Complex 4: O_2_ + 4cyt_c2 + 8H ⇔ 4cyt_c3 + 2H_2_O + 4H**

Kcf_C4 = 93.5;

Ks_C4 = 110E-6;

Complex4_reaction = (Vmax_C4 * Kcf_C4*Cyt_c2) / (Ks_C4 + Cyt_c2);

**Complex 5: ADP + Pi + 3H ⇔ ATP + H_2_O + 3H**

Kcf_C5= 14.5;

Kd_C5 = 2.67E-7;

Kp_C5 = 9.02E-5;

Kt_C5 = 4.33E-5;

Khx_C5= 1.3E-4;

Khy_C5= 1.6E-4;

Kltf_C5 = 1.35E8;

Kltr_C5 = 0.00018;

ax_C5 = 0.1;

ay_C5 = 0.6;

b_C5 = 0.3;

t_C5 = 310;

num5 = (((ADP*mPi)/(Kd_C5*Kp_C5))*Kltf_C5*(cH/mH)^(-3*(b_C5-ax_C5))...

*(cH/(Khx_C5*((cH/mH)^ax_C5)))^3) ...

- (((ADP)/(Kt_C5*Kp_C5))*Kltr_C5*(cH/mH)^(-3*(1-b_C5-ay_C5))...

*(mH/(Khx_C5*((cH/mH)^-ay_C5)))^3);

denom5 = ((1 + (cH / (Khx_C5*(cH/mH)^ax_C5)) + (mH / (Khy_C5*(cH/mH)^-ay_C5)))^3)* (3+ ((ADP*mPi)/Kd_C5*Kp_C5) + (ATP/Kt_C5));

Complex5_reaction = (Kcf_C5*Vmax_C5*num5) / denom5;

**Differential equations to be solved**

Rate of Glucose

dydt(1,1) = (( GLUT_reaction - GK_reaction - ALDR_reaction));

Rate of ATP

dydt(2,1) = (- 2*GK_reaction - PFK1_reaction + PGK_reaction + PYK_reaction - ATPase_reaction - AK_reaction + 13*OxPhos_reaction + Complex5_reaction);

Rate of G6P

dydt(3,1) = (GK_reaction - HPI_reaction - G6PD_reaction);

Rate of ADP generation

dydt(4,1) = (2*GK_reaction + PFK1_reaction - PGK_reaction - PYK_reaction + ATPase_reaction + 2*AK_reaction - 13*OxPhos_reaction - Complex5_reaction);

Rate of F6P

dydt(5,1) = (HPI_reaction - PFK1_reaction + TA_reaction + TK2_reaction + HK_reaction);

Rate of FBP

dydt(6,1) = (PFK1_reaction - ALDO_reaction);

Rate of DHAP

dydt(7,1) = (ALDO_reaction - TPI_reaction);

Rate of G3P

dydt(8,1) = (ALDO_reaction + TPI_reaction + TK1_reaction + TK2_reaction - TA_reaction - GAPDH_reaction);

Rate of NAD

dydt(9,1) = (- GAPDH_reaction + LDH_reaction + DHAses_reaction + MDH1_reaction - SoDH_reaction - Complex1_reaction);

Rate of 13BPG

dydt(10,1) = (GAPDH_reaction - PGK_reaction);

Rate of 3PG

dydt(11,1) = (PGK_reaction - PGAM_reaction);

Rate of 2PG

dydt(12,1) = (PGAM_reaction - ENO_reaction);

Rate of PEP

dydt(13,1) = (ENO_reaction - PYK_reaction);

Rate of Pyruvate

dydt(14,1) = (PYK_reaction - LDH_reaction - PYRH_reaction + CMALIC_reaction);

Rate of Lactate

dydt(15,1) = (LDH_reaction - MCT_reaction);

Rate of AMP

dydt(16,1) = ( - AK_reaction);

Concentration of 6PG

dydt(17,1) = (G6PD_reaction - sixPGDH_reaction);

Concentration of Ru5P

dydt(18,1) = (sixPGDH_reaction - RPE_reaction - RPI_reaction);

Concentration of Xyl5P

dydt(19,1) = (RPE_reaction - TK1_reaction - TK2_reaction);

Concentration of R5P

dydt(20,1) = (RPI_reaction - PRPPS_reaction - TK1_reaction);

Concentration of E4P

dydt(21,1) = (TA_reaction - TK2_reaction);

Concentration of S7P

dydt(22,1) = (TK1_reaction - TA_reaction);

Concentration of NADP

dydt(23,1) = (GSSGR_reaction - G6PD_reaction - sixPGDH_reaction - CMALIC_reaction - cIDH_reaction + ALDR_reaction);

Concentration of GSH

dydt(24,1) = (GSSGR_reaction - GPX_reaction);

Concentration of Mitochondrial Pyruvate:Pyrm

dydt(25,1) = (PYRH_reaction*(Vc/Vm) - PDH_reaction + MMALIC_reaction - PC_reaction + GPT_reaction);

Concentration of mAcCoA

dydt(26,1) = (PDH_reaction - CS_reaction);

Concentration of Citrate in the mitochondria, mCIT

dydt(27,1) = (CS_reaction - ACON_reaction + CITMAL_reaction);

Concentration of mitochondrial Isocitrate

dydt(28,1) = (ACON_reaction - IDH_reaction + ISOCITMAL_reaction);

Concentration of Alpha-ketoglutarate

dydt(29,1) = (IDH_reaction - AKGD_reaction - GOT2_reaction + AKGMAL_reaction + GDH_reaction - GPT_reaction);

Concentration of Mitochondrial SCoA

dydt(30,1) = (AKGD_reaction - SCOAS_reaction);

Concentration of Mitochondrial Succinate

dydt(31,1) = (SCOAS_reaction - SDH_reaction);

Concentration of Mitochondrial Fumarate

dydt(32,1) = (SDH_reaction - FUM_reaction);

Concentration of Mitochondrial Malate

dydt(33,1) = (FUM_reaction - MDH2_reaction - AKGMAL_reaction - CITMAL_reaction + MALPi_reaction - MMALIC_reaction - ISOCITMAL_reaction);

Concentration of Mitochondrial Oxalaacetate

dydt(34,1) = ( - CS_reaction + MDH2_reaction + GOT2_reaction + PC_reaction);

Concnetration of Mitochondrial Aspartate

dydt(35,1) = (ASPGLU_reaction - GOT2_reaction);

Concentration of Mitochondrial Glutamate

dydt(36,1) = (GOT2_reaction - ASPGLU_reaction + GLUH_reaction - GDH_reaction + GPT_reaction);

Cytosolic aspartate

dydt(37,1) = ( - GOT1_reaction - ASPGLU_reaction*(Vm/Vc));

Cytosolic glutamate

dydt(38,1) = (GOT1_reaction + ASPGLU_reaction*(Vm/Vc) - GLUH_reaction*(Vm/Vc) + GLS_reaction);

Cytosolic oxaloacetate

dydt(39,1) = (GOT1_reaction - MDH1_reaction + CLY_reaction);

Cytosolic Malate

dydt(40,1) = (MDH1_reaction + AKGMAL_reaction*(Vm/Vc) + CITMAL_reaction*(Vm/Vc) + ISOCITMAL_reaction*(Vm/Vc) - MALPi_reaction*(Vm/Vc) - CMALIC_reaction);

Cytosolic AKG

dydt(41,1) = ( - GOT1_reaction - AKGMAL_reaction*(Vm/Vc) + cIDH_reaction);

Cytosolic Citrate:cCIT

dydt(42,1) = ( - CITMAL_reaction*(Vm/Vc) - CLY_reaction - Aconitase2_reaction);

Rate of Glutamine in the cytosol

dydt(43,1) = (ASCT2_reaction - GLS_reaction);

Rate of NADH

dydt(44,1) = (GAPDH_reaction - LDH_reaction - DHAses_reaction - MDH1_reaction + SoDH_reaction + Complex1_reaction);

Concentration of NADPH

dydt(45,1) = ( - GSSGR_reaction + G6PD_reaction + sixPGDH_reaction + CMALIC_reaction + cIDH_reaction - ALDR_reaction);

Concentration of GSH

dydt(46,1) = ( - GSSGR_reaction + GPX_reaction);

Concentration of O2i

dydt(47,1) = (Oxtransfer_reaction);

mGDP

dydt(48,1) = (- SCOAS_reaction);

mGTP

dydt(49,1) = (SCOAS_reaction);

mALA

dydt(50,1) = (- GPT_reaction);

cICIT

dydt(51,1) = (Aconitase2_reaction - cIDH_reaction - ISOCITMAL_reaction*(Vm/Vc));

Lac_out

dydt(52,1) = MCT_reaction;

Glutamine_out

dydt(53,1) = - ASCT2_reaction;

O2e

dydt(54,1) = - Oxtransfer_reaction;

Fru

dydt(55,1) = SoDH_reaction - VfruT - HK_reaction;

Sor

dydt(56,1) = ALDR_reaction - SoDH_reaction;

Cyt 3+

dydt(57,1) = -2*Complex3_reaction + 4*Complex4_reaction;

Cyt 2+

dydt(58,1) = 2*Complex3_reaction -4*Complex4_reaction
