# Supplementary material for: Kinetic and data-driven modeling of pancreatic β-cell central carbon metabolism and insulin secretion
Source: PLoS Comput Biol. 2022 Oct 17;18(10):e1010555. doi: 10.1371/journal.pcbi.1010555 (PMC9612825; doi:10.1371/journal.pcbi.1010555)
Supplement: Attachment — Submitted filename: Response to Reviewers_Final.pdf [file pcbi.1010555.s008.pdf]

Response to Reviewers

We give great thanks to the reviewers for their constructive review. Below, we have provided a response to each comment, written in blue font, and indicated how we have edited the manuscript text to address the comments. Additionally, we have provided a file that highlights the changes made to the manuscript text; the line numbers below refer to that file (named: "Manuscript_R1_trackChanges.docx").

Reviewer #1:
Diabetes is a common chronic disease manifesting when not enough insulin is produced by the pancreatic beta-cells. Although several efforts have been made within the community to understand the metabolism of these cells in response to high glucose, up to date, b-cells metabolism is not well characterized. In this work, the authors developed a computational modeling workflow that includes a comprehensive model for beta-cells expanding existing knowledge, data integration, parameter identification, and model simulations. With this model, the authors were able to study the metabolic sensitivity of these cells to glucose and simulate the effect of used drugs. The analysis presented here can be used to guide experimental design and drug development. Therefore, the content of this manuscript is highly relevant to the field.
We appreciate the reviewer's support of the study. We have addressed the comments below to improve the manuscript.

Major comments:
1.Given the importance of energy in the metabolism of beta-cells, the authors could comment why they have decided not to include the ETC reactions in their model.
We thank the reviewer for this point, and agree that the electron transport chain is an important component of the modeled system. In the original submission, we unintentionally neglected, by just using an existing model of pancreatic beta cell metabolism. We have added the ETC, re-parameterized the model, and updated all simulations and analyses. We truly thank the reviewer for raising this point – though the main conclusions are not substantially different from the prior iteration, the model is both more complete and more accurate in its predictions.

2.In figure 1, it is mentioned that the reaction equations are given in the supplementary material; however, I could not find this information.
We thank the reviewer for this comment and apologize for the oversight, the set of reaction equations has been added to the supplemental information. The full model is also available on our GitHub repository, as noted in the "Data Availability and Supplementary Material" section.

3.In section 2.2, the authors say that the model contains a total of 56 metabolites and 61 reactions. However, only 39 metabolites (12glycolysis + 7ppp + 20tca) and 45 reactions (13 glycolysis + 7 ppp + 25tca) are presented in the main text. It would be nice that the authors specify in the text which reactions were associated to the polyol pathway and also elaborate on the rest of the reactions that are part of the network.
We thank the reviewer for the comment. We have added within the main text explicit mention of the metabolites and reactions in the polyol pathway (Lines 196-197) and reactions that were not part of the glycolytic, PPP, TCA cycle, polyol, or ETC (Lines 218-223), rather than simply providing the figure showing those model components.

4.In line 206, it is not clear why the model contains 92 reaction velocities (Vmax) if there are 61 reactions in the network. The authors can consider describing the type of the rest of the 385 parameters. Furthermore, the authors should explain what are the directionalities considered for the reactions.

Thank you for mentioning this potential source of confusion. Because there are some reaction rate expressions that have both $V_{max,f}$ and $V_{max,r}$ values, there are more reaction velocities than modeled reactions. We have clarified this point in the manuscript by replacing instances of "Vmax" with the more informative or inclusive term "reaction velocity". We have also added more detail about the remaining (non-V_{max}) parameters that are in the model but not varied. These edits are provided in Lines 233-238.

5.Line 251, it is not clear to which parameters they are referring. If it is the case, the authors can explicitly write "correlated to their corresponding v_F". It may be helpful to explain the nomenclature used for Vmax, v_F and v_R, which of them represent parameters, and which flux values.
We thank the reviewer for mentioning this point. Because of the nonlinear and dynamic nature of the model, it is possible for any two model parameters to be correlated, and therefore be structurally unidentifiable; however, for our model, the only reactions found to be correlated to each other were Vf/Vr pairs for the same reaction. We have clarified this point in the text in lines 290-293.

6.In Figure 5, the authors write that all the Vmax are depicted, but there are only 33 reactions in the plot. Are these chosen reactions? In this graph, what does the red star next to some reaction names represent?
We have corrected this point, as the figure shows the Vmax values which, when varied, brought about a change in metabolite levels; the reaction velocities that did not affect any metabolite fold change were excluded from the figure for increased readability. The starred reactions were those that the PLSR analysis identified as influential. We have edited the manuscript in line 594 to address these points.

7.Line 539, in the figure, only the case of increased flux is shown.
The left panel shows the decrease, and the right panel shows the increase. We have changed the labeling from "knockdown and increase" to "decrease and increase" in order to reduce this potential confusion.

8.Is there any evidence in the literature supporting the results for section 3.3? Even if it is partial, adding this would improve the impact of the model.
We appreciate the reviewer raising this point. Unfortunately, there have not been many metabolomics analyses that perturb reactions or reaction rates, as most assess the impact of supplementing with extracellular metabolites or drugs. However, we were able to find some very relevant experimental studies:

- Hasan et al suppressed *pyruvate carboxylase (pc)* reaction activity in INS1 cells:
NADPH levels were unchanged (consistent with our model)
Pyruvate levels increased (consistent with our model)
Lactate levels increased (unchanged in our model)
Malate levels decreased (consistent with our model)
Citrate levels decreased (consistent with our model)

- Jensen et al showed *pc* knockdown had no effect on NADPH/NADP ratio, as consistent with our model

- Guay et al showed knockdown of the Malic enzyme caused a decrease in glucose oxidation (the steps converting glucose to pyruvate). The model also predicts a decrease in glycolytic intermediates (1,3BPG, 2PG, 3PG, PEP) upon suppression of malic enzyme.

- MacDonald et al show the *idh* knockdown changes the NADPH/NADP ratio; however, our model does not show *idh* changing metabolite levels when decreased.

We have added a description of these results to the Discussion section to place our work in context and compare to existing studies (Lines 807-816).

9.In figure S3, it is not clear why the flux through the reactions gssgr and gpx is increased but not the flux through ak.
We thank the reviewer for his comment; the modeled results indeed show the *ak* reaction flux increases (as it should, since that is the perturbation implemented); we have corrected this error in the figure and have also made the *ak* reaction label bolded, to emphasize that it is the reaction being perturbed (in the revised manuscript, this is now Figure S5). Similarly, the *g6pd* reaction label was bolded in Figure 7 to show that it was the perturbation being performed.

10.The authors should consider creating a repository with the code and data necessary to ensure the reproducibility of the results of the paper. I could not find any reference to the code in the current manuscript.
A GitHub repository was referenced in the manuscript's method section (line 114 of the original submission). We have moved this reference to the end of the paper, in the "Data Availability and Supplementary Material" section, to be more easily found and accessed. We have also provided in the supplement the original collected data for added transparency, rather than simply referencing the sources.

Minor:
1.Line 232 typo "the"
We fixed the typo, changing "he" to "the".
2.Line 527-528 redundant "perturbing" "perturbed"
We thank the reviewer for noticing this; we removed the clause containing the second "perturb"

Reviewer #2:
The manuscript by Gelbach and co-workers describes a computational model based on ordinary differential equations of the pancreatic beta cell carbon metabolism formulated as a tool to study the glucose stimulated insulin secretion. In general, the model is presented as very accurate although the reader has no means to judge or contrast this claim. In fact, the results presented are mainly qualitative in terms of biological significant information that can be contrasted with different sets of experimental data.
Thank you for your time and effort evaluating our paper, and we appreciate the critique of the work. We have expanded the presentation of the manuscript to better emphasize the quantitative nature of the data used for model training and validation, and to include additional types of data to further support the model's utility.

Model and data presentation:
On the one hand there is a careful and detailed description of the methods used for adjustment (model training) of parameters and sensitivity analysis of the main parameters such as Vmax. On the other hand, the authors do not present neither the model kinetic equations nor the code used in their algorithms.
We thank the reviewer for these points. A GitHub repository was referenced in the manuscript's method section (Line 114 of the original manuscript) and has now been moved to the end of the paper to be more easily found and accessed (in the "Data Availability and Supplementary

Material" section). We now include additional detail in the supplement describing the model reaction equations, parameter types and values, and the naming/abbreviations for all model components. We have also provided the original collected (experimental) data for added transparency, rather than simply referencing the sources.

Also, no model simulated flux values or the so much advertised by the Authors flux distributions are shown, that could be contrasted with real fluxes in any model. For example, mitochondrial respiration measurements can be used to estimate the TCA cycle flux in cells, and be used to compare the simulated flux values. In such scenario, the usefulness of this manuscript is very limited and not a real contribution that could benefit the field.
We appreciate this point, as we acknowledge that the model's predicted flux values are a major point of the paper that lacked sufficient detail or data. We now provide the flux distribution upon stimulation with 16.7mM glucose, shown as Figure 2B. Additionally, we have found available published data measuring the flux through select reactions, and we added a comparison of those data with model predictions, in the new Supplementary Figure S4.

I was surprised by the plots in Figures 6 and 7 displaying only qualitative results in the form of colors of intermediates concentrations.
We believe that the figures demonstrate the systems-level changes in predicted metabolite amount and fluxes and enable the reader to quickly see the network-level changes due to glucose stimulation and the implemented interventions. However, we acknowledge that the figures do not show quantitative differences between the treated and base conditions. In order to address this, we have added Table 1 to the main text, which presents the effect of the metabolic perturbations on measurements predicted to be impactful in beta cells, including effects on insulin, the NADPH/NADP ratio, ATP/ADP ratio, and pyruvate cycling. We have also added tables to the supplement giving readers direct access to the data used to make these figures.

Another important variable value is related to the redox state in the various conditions. The authors mention increases in NADH, NAD and glutathione but fail to mention and compare the state of the redox couples (NADH/NAD). The importance of this metric is the abundance of biological data under different experimental condition that could be used to validate this model.
This is a great point; we are grateful that the reviewer raised it. Though both the NADH/NAD and NADPH/NADP ratio are believed to be promising markers of redox state, the NADPH/NADP ratio is viewed as most important in the beta cell.
We have therefore added additional detail on the NADPH/NADP ratio to the model predictions presented. For the base predictions (treatment with glucose only - sections 2.5 and 3.1), we have added literature support from Spegel et al. that was used for training and validation in our manuscript, and mention the comparison in lines 491-496. We also include the NADPH/NADP and NADH/NADH redox pairs in the quantitative descriptions of each metabolic perturbation (in section 3.5) as Table 1.

Given the objective of searching for new targets to treat (type 1) diabetes, the reliability of the biological assumptions behind the model equations and the goodness of the comparison of biological variables is fundamental. Without that possibility of contrasting the modeling results, the model is not useful.
We thank the reviewer for his points, and agree that we can increase the utility of and confidence in the model by providing detailed measurements verifying the model. In addition to our previously presented validation data for model training (Figure 1 for qualitative metabolite

changes and Figure 2 for quantitative changes) and the presented references and information in the manuscript's discussion section, we now include additional data and reference points:
- For predictions of metabolite fold changes, we now demonstrate a more concrete and statistical agreement with the data through a series of t-tests, given in the supplement and mentioned in section 3.1. (Lines 466-471)
- For the predictions of flux values, we now present the model predictions as Figure 2B, which can be tested by future research. Despite relatively limited availability of data that explicitly measures the flux through metabolic reactions, we have found and present validation for flux predictions as shown in Supplementary Figure S4. These results are discussed in Lines 498-505.
- For the kinetic model predictions of metabolic perturbations, we have expanded the discussion section (4.1) to explicitly mention published data showing the effect of *in vitro* knockdowns on metabolite levels, thus providing explicit support for the model predictions (Lines 807-816).

We believe these additions greatly strengthen the paper and its utility.

Figures comments:
Figure 1: The color scheme chosen provides very bad contrast in several metabolites which added to the low resolution of the pdf document render this scheme rather unreadable. The lettering should be clearly contrasted with the background for readability.
We thank the reviewer for the comment. We have adjusted the metabolite and label coloring for increased clarity.

Figure 2. The scale of ordinates is logarithmic. I understand that this is due to the orders of magnitude difference between the various metabolites. However, the error bars do not mean much in a logarithmic scale and the difference between computed and experimental data are downplayed with such a scale. Instead of showing the plot, I suggest adding a table with the real values together with standard errors. Alternatively, a plot in linear scale could give a more quantitative appreciation of the "goodness" of the simulation results with the computational algorithm.
We thank the reviewer for the comment, and have adjusted the y-axis scale to be linear.

Figure 3. The data are normalized. However, in this way the reader cannot compare the simulations to experimental data. On the one hand, no information about the units that are being used (specifically in Figure 3B). This is completely abstract presentation not appropriate for a journal with a large biological audience.
We thank the reviewer for this comment. We have revised Figure 3 as described below.
- To help the reader better take in the model predictions, we have changed the y-axis in panel A to state "Percent Variance Explained", and therefore the scale to go from 0-100.
- Regarding panel B, we note that a customary preprocessing step to properly run partial least squares regression analyses is to standardize or normalize of the data, which we have done and had shown in the original manuscript. In the revised manuscript, we back-calculated from the normalized output (both experimental data, x-axis, and PLSR model predictions, y-axis) to present the results in a way that is easier to understand biologically and to compare to other data. The figure shows the fold change for insulin, for the high glucose condition relative to the basal condition (16.7mM/2.8mM). Because of this, the output is unitless.
- The PLSR model weights and VIP scores, for panels C and D respectively, are both unitless measurements. We have added this point to the methods section 2.6 (Line 368).

Figure 5: There is no indication of the color scale? Additionally, the presentation is rather problematic, since most representations display the "adjusted" variable in the x axis and the resulting (e.g. the change in metabolite level) in the y-axis, which is the opposite display in this figure.
We have enlarged the color scale to make it clearer and more visible. We have also flipped the orientation of the figure, to make the dependent and independent variables more intuitive.

Minor points:
Line 101: What do you mean by "no sufficient model"? Please, rephrase.
We have edited the manuscript text to state “there is a current need for greater understanding of core central carbon metabolic pathways and how their dynamics correlate to insulin secretion.” (Lines 103-105)

Line 232: typo “he” instead of “the”
We fixed the typo, changing “he” to “the”.

Line 241: Clarify the origin of the “training” dataset: Spegel et al or Malmgren et al or both?
We appreciate this comment, and have added a sentence to further clarify that the training dataset was the combined Spegel data (3- and 6-minute time points) and the Malmgren (60-minute timepoint), line 274-276.

Line 249. Pairwise parameter identifiability analysis is jargon. Please, explain.
Thank you for this point. We have adjusted the sentence to read “In order to properly fit the kinetic model, we first performed an *a priori* parameter identifiability analysis. Specifically, we varied each model reaction rate to determine which parameter pairs may be mathematically correlated to each other and therefore structurally non-identifiable”. Furthermore, we have now provided additional citations to explain the concept of and mathematics behind parameter identifiability in greater detail." (Lines 282-285)

Line 677 Diabetes type 1 I guess is what the authors are referring to…
We thank the reviewer for pointing this out, as it is an important distinction to make. Though a greater understanding of beta cell metabolism can potentially inform treatment for both types, our modeling work is more appropriately oriented towards Type 2 diabetes. This is because T1DM is seen as an immune mediated, while beta cell dysfunction in T2DM is seen as metabolic in nature. We have clarified this in the manuscript (Line 785).

Line 705 glutathione, reduced or oxidized?
We have clarified that the reduced form of glutathione (GSH) is the antioxidant of interest (Line 829).

Line 730. Why is a model limitation not considering the heterogeneity of the lack of interactions with other cell types? Which would be the consequences of considering such interactions? Or the cellular heterogeneity?
The reviewer raises an important point, and we have edited the discussion to include this limitation. *In vivo,* pancreatic beta cells exist and operate with many other cell types in the pancreas and many different secreted metabolites or factors in the microenvironment; for example, alpha cells and beta cells interact in a paracrine manner, with beta cells releasing insulin (thus lowering blood glucose levels) and alpha cells releasing glucagon (increasing blood glucose levels). The model we developed, and the data available to inform model parameter values and predictions, sought to explore a simplified case, focusing on beta cell activity alone.

This simplification is intrinsically a limitation of the model. We have edited the discussion to more explicitly mention this limitation (Lines 854-857).

Reviewer #3:

Summary

The authors have constructed a kinetic model for central carbon metabolism in pancreatic beta cells using previously published intracellular metabolite concentration data. This kinetic model is then used to generate flux distributions, which are correlated with insulin secretion using a partial least-squares model. The authors have used this model to predict the pharmacological effects of commonly prescribed anti-diabetic drugs such as metformin and identify novel targets that can be modulated to enhance insulin secretion in the beta cells.

We appreciate the reviewer's time in assessing our work.

Major comments:

1. The authors have parameterized the kinetic model for central metabolism after identifying influential parameters using the eFAST method. The authors must parameterize the complete kinetic model using particle swarm optimization to justify their assumption that their neglected parameters are indeed not influential. This is important because the authors have identified distinct kinetic models that explain the metabolomics data to the same extent. Poor resolvability of models could also suggest data insufficiency.

We thank the reviewer for raising this point, and agree that testing the results of the sensitivity analysis by fitting the entire set of model parameters is valuable. We have performed particle swarm optimization on the full model and confirmed that the full model performs similarly to the model with only parameters that had been predicted to be impactful. However, we moved forward with the fitting obtained by estimating the influential parameters as in that case, the estimated parameter values were within a close range rather than being non-identifiable. We have added a statement of these findings in Lines 324-330.

2. The authors must perform a more rigorous statistical analysis that includes a χ^2-goodness of fit to demonstrate that the parameterized kinetic model is statistically significant.

We thank the reviewer for raising this point, and recognize that concrete statistical analysis of the model is needed. We believe that the t-test is the appropriate statistical approach for testing data points to determine if they are from the same distribution. We performed multiple t-tests, comparing each model fold change prediction with its corresponding experimental data point. That gave the result that nearly all the model predictions and data are not statistically significantly different, meaning that they match well. We have included this in section 3.1, in lines 466-471, and provide the raw results of the analysis in the supplement (Table S1).

3. The experimental data shown in figure 2 is plotted in logarithmic scale, suggesting the coefficient of variation in the experimental data is very large. The authors must comment on how this would impact the variability in flux predictions in their eight parameterized models.

That is correct, as the fold change measurements for metabolites ranged over three orders of magnitude. We believed that we had accounted for this with our fitting technique's objective function, wherein each data point's error calculation (the difference between the model prediction and experimental data) was scaled by the data's standard deviation (to preferentially fit the high-confidence data) and by the data's magnitude (to avoid prioritizing the large fold change metabolites at the expense of the smaller data points). That approach enabled us to fit the entire dataset and derive a consistent and high-confidence set of parameterized models.

However, we certainly agree that we had not provided enough information on this point in the original manuscript submission. Explicit mention of this has been added to Lines 313-316.

4. The low Q^2 Y values reported during PLS have been attributed to the size of the dataset (only five time points). Because the model is constructed to modulate insulin secretion, additional data is needed to validate the interesting hypotheses generated in section 3.2. In particular, the authors must provide additional evidence to substantiate their associations between adenylate kinase and TCA cycle activity on insulin secretion.
Thank you for this point, as we neglected to include support in the discussion section for those predictions in the original manuscript submission. We have now provided additional discussion (and referenced published literature) to support and validate the model predictions, given in lines 761-775.

5. It is surprising to note that perturbing the Vmax parameter of most reactions has little to no effect on the metabolites from pentose phosphate pathway and redox metabolism. The authors must explain why this occurs in the context of their kinetic model. Were the enzyme saturation levels too low due to a very large K_M?
We thank the reviewer for making this point. We do not believe that the enzyme saturation levels were too low, as pentose phosphate pathway Km values were of similar magnitude to those of other pathways in the model. Rather, we believe that the PPP's minimal activity in beta cells (observed experimentally and in our model) causes the pathway to be relatively robust to perturbations.

Minor comments:
In line 82, the authors claim that "measurements of metabolite pool sizes do not give insight into the cellular dynamics or kinetics…". Since fluxes are directly related to changes in the metabolome, the authors' claim may not be accurate. It is recommended that the authors reword this claim to make a case for constructing a kinetic model.
We thank the reviewer for this comment. We have adjusted the sentence: "Because measurements of metabolite pool sizes alone do not give holistic insight into cellular behavior, their utility may be extended by integrating the data into frameworks that predict transient dynamics, thus linking experimental measurements with phenotypic understanding." (Lines 81-84
