# Supplementary material for: Kinetic and data-driven modeling of pancreatic β-cell central carbon metabolism and insulin secretion
Source: PLoS Comput Biol. 2022 Oct 17;18(10):e1010555. doi: 10.1371/journal.pcbi.1010555 (PMC9612825; doi:10.1371/journal.pcbi.1010555)
Supplement: Attachment — Submitted filename: ResponseToReviewers_R2.docx [file pcbi.1010555.s009.docx]

**Response to Reviewers**

We appreciate the reviewers for their constructive comments on the revised manuscript. Below, we have provided a response to each comment, written in blue font, and indicated how we have edited the manuscript text to address the comments. Additionally, we have provided a file that highlights the changes made to the manuscript text; the line numbers below refer to that file (named: “Manuscript_R2_trackChanges.docx”).

**Reviewer #1:**

The authors have addressed all my comments.

We appreciate the reviewer’s support of our work, and again thank them for their effort and guidance in improving the manuscript.

**Reviewer #2:**

I have evaluated the response of the authors to the specific points I raised with respect to the original manuscript. I found the model formulation and results rather disappointing both computationally and from a biological standpoint. The main issue is that this model is not able to evolve to a steady state, and the only quantitative data shown in Figure 2B really does not make any biological sense, likely because of an ill-designed model. There are no mass conservations relations. There are sinks and sources of intermediary metabolites not accounted for, or there is accumulation or depletion of metabolites that are not appropriately described in the manuscript.

We appreciate the reviewer’s point regarding formulation of the metabolic network and mass conservation. We agree that previous versions of the manuscript did not provide sufficient detail regarding these two points. However, this is a lack of detail in the manuscript rather than an ill-designed model. Thus, we have revised the manuscript to clarify these points. We now explicitly mention in Section 2.1 of the Methods which reactions are sources and sinks (Lines 162-164 and 186-187). Furthermore, we provide the stoichiometric matrix of the metabolic network (Supplementary Table S1). With the stoichiometric matrix, we demonstrate that the network is indeed mass-balanced, and we identify the metabolites that constitute conserved pools. This information is given in Section 2.2 of the Methods (Lines 239-243). We believe this gives clarity on the model’s conservation of mass and its overall suitability for studying pancreatic beta cells.

As described in the equations that the authors now added in response to my request to their first submission, this model won’t reach a steady state. A steady state behavior is important for several reasons: 1) the need to know where in the parametric space the model behavior maps and to distinguish between steady and transient states; 2) from a steady state, the modeler can introduce a perturbation to know whether the model behavior returns to the state before the perturbation or if it evolves to another steady state, if not then the model is in an unstable region of the parametric state and this should be known to distinguish from transient states; 3) if using the model to simulate a flux distribution throughout a metabolic pathway, it should be known whether the parameterization makes any biological sense because, in principle, the space of solutions of a model is big and the key biological question is to which solution in that space belongs the experimental data that the model is trying to simulate. In other words, metabolic networks in biological systems typically show features of stability of the internal medium (homeostasis/homeodynamics) and this should be reflected by the model’s steady state. It is also important to demonstrate its robustness against alterations in the environment which will again be reflected by the ability of the system to return to the original state after a perturbation. The condition of steady state will not show, of course, at the point of a perturbation (e.g. glucose concentration increase point from 2.8 to 16.7 mM), but the simulations of the metabolic behavior should come back to a steady state and the authors have failed to show a temporal evolution toward a steady state. Instead, they show averages of time points during the perturbation. I believe the authors need to improve the design of the model as applied to pancreatic beta-cells and use the model to answer meaningful questions.

The reviewer is certainly correct that it is vital for the system to show stability and to reach a steady state. We acknowledge that we had not sufficiently considered this in the previous model fitting, as we solely used experimental data for the relative levels of metabolites: we fit the model to the metabolite fold changes of a high glucose (16.7mM) relative to low glucose (2.8mM) condition. However, this information alone is not sufficient to constrain the predicted metabolite levels in both conditions from increasing monotonically, even if the model accurately captures the fold change measured experimentally. To fix this, we uncovered an additional dataset and used it in the model fitting approach. Specifically, Spegel et al. (ref. 54 in the manuscript) provided metabolite levels relative to the 0-minute time point. By using these data for model fitting, we are able to constrain the model predictions and ensure the fitted parameter sets exist in a biologically reasonable solution space, including a reasonable steady state. We display the steady state produced by the model in Figure 3, which shows the long-term dynamics of the model predictions for metabolites measured by Spegel, wherein each metabolite reaches a steady state level. In addition, we added Supplementary Figure S4, which shows the predicted timecourses for all metabolites included in the model (displayed as absolute concentrations).

**Reviewer #3:**

The authors have done a very good job addressing all of my previous comments and I will be excited to see this work published.

We appreciate the reviewer’s support of our work, and again thank them for their effort and guidance in improving the manuscript.
