# Supplementary material for: Kinetic and data-driven modeling of pancreatic β-cell central carbon metabolism and insulin secretion
Source: PLoS Comput Biol. 2022 Oct 17;18(10):e1010555. doi: 10.1371/journal.pcbi.1010555 (PMC9612825; doi:10.1371/journal.pcbi.1010555)
Supplement: Attachment — Submitted filename: Response to reviewers_R3.docx [file pcbi.1010555.s010.docx]

**Response to Reviewers**

We appreciate the reviewers for their constructive comments on the revised manuscript. Below, we have provided a response to each comment, written in blue font, and indicated how we have edited the manuscript text to address the comments. Additionally, we have provided a file that highlights the changes made to the manuscript text; the line numbers below refer to that file (named: “Manuscript_R3_trackChanges.docx”).

**Reviewer #1:**

The authors had addressed all my comments.

**Reviewer #2:**

After two rounds of evaluation of the manuscript by Gelbach et al. I can state that the main problem is the presentation of the model and the results. The authors have shown that the model is able to achieve a steady state at 16.7mM Glucose (but they have not shown that as well for the condition at 2.8 mM glucose). Also, they show through the stoichiometric matrix that the model is well balanced. However, there are still numerous issues that should still be clarified.

Concerning the model, each of the 65 enzyme rate expressions has many parameters, in addition to those 85 (although the authors state 81 in the main text) that need to be adjusted following the experimental data available (not for every metabolite) of 56 metabolites.

We appreciate the reviewer noticing the typo – it is indeed 85 Vmax parameters. We have corrected this error.

We note that we do not fit all 85 Vmax parameters; instead, we selected to fit the 33 Vmax parameters that emerged as most influential in the eFAST global sensitivity analysis. Following the paradigm of developing kinetic ODE models, we determine which parameters the model predictions are most sensitive to and fit only those influential parameters. In doing so, we produce a model that is calibrated to experimental data and attempt to avoid overfitting. We have added additional information in the Methods to clarify this point (Lines 323-326).

The reviewer is correct that the reaction rate expressions include many other parameters, which we do not fit. It is not common practice to fit every parameter in a model, as it leads to parameter identifiability problems. In addition, previous work suggests that fitting the reaction velocities (forward and reverse Vmax values) is sufficient to develop a predictive of metabolism that reflects cellular behavior^2,3,6^. We apply this consideration to reduce the number of fitted parameters and aim for identifiable parameters. We edited the manuscript to mention this point in section 2.2 of the Methods (Line 240).

We acknowledge that we do not have experimental data for each metabolite in the model. This is most frequently the case in developing ODE models – that the model predicts quantities that have not been measured experimentally. We believe this is an advantage of computational modeling such that more quantities and conditions can be simulated, complementing experimental studies. However, we are quite fortunate in this study to have 70 experimental data points (from multiple published papers, at various time points) that we can use to estimate the values of the 33 most influential Vmax values. We believe the available data is sufficient for producing a calibrated predictive model. We describe that data collection in section 2.4 of the Methods and explicitly state the number of data points available for fitting (Lines 295-297).

Not only the value, but also the units and source of parameters should be stated in the supplementary information.

We agree that for each model parameter, it is important to list the value, units, and literature reference. Though we described the sources for the model equations and parameters for each pathway in the Methods (Section 2.2), it was an oversight on our part to not explicitly provide the units and references in a single table. The revised submission now includes the parameter values, units, and source in Table S5 (a new addition for this revised version of the paper).

This reviewer wonders if there is enough data to constraint such a large parameter sets or if the system is underdetermined? And what is the method used to constraint that large set of parameters?

As mentioned above, we have found 70 experimental data points from multiple published papers and at various time points that we can use to estimate the values of the 33 most influential Vmax values. We believe that the model is reasonably well constrained for two reasons: (1) the number of data points used for model fitting exceeds the number of fitted parameters, and (2) we obtain relatively consistent sets of estimated parameters from the best fits (Figure S3). The revised manuscript now explicitly mentions the number of data points used in training and validation (Lines 295-297). We now also emphasize that we account for variability in parameters by repeating the model fitting multiple times, aiming to arrive at parameters that represent a global minimum for our error function (Lines 349-351).

We describe the method used for parameter fitting in Section 2.5 of the Methods. The steps are described below as well.

Step 1 (Line 304-315): We use an *a priori* parameter identifiability analysis to determine mathematically correlated parameters, and we relate correlated forward and reverse Vmax parameters to each other with an equilibrium constant, *K*. This ensures that we do not simultaneously fit both forward and reverse reaction velocities.

Step 2 (Lines 317-328): We perform an eFAST global sensitivity analysis of the model parameters, while simulating the same conditions (glucose concentration and stimulation time) as the experimental data to be used for parameter fitting.

Step 3 (Lines 330-351): We perform particle swarm optimization (PSO), simulating the same experimental conditions from the published studies that we extracted data used for parameter fitting. We allow the parameters to vary 100-fold above and below their starting point. That user-imposed range further constrains the estimated parameters. From this analysis, we determined the subset of Vmax parameters that the model predictions are most sensitive to. Those influential parameters are used for fitting.

It is not clear how the authors discriminate experimental data on metabolites that are present both in mitochondria and cytosol (shared metabolites). The authors state that there is only one pool of metabolites detected by MS, but figure 1 shows concomitant changes in model and experiment for most shared metabolites

We do not split the data; rather, we fit the shared metabolites to the shared single pool, comparing the measured data to a sum of the two equations, weighted by relative compartment volume. We added more detail in the Methods to clarify this point (Lines 275-278). We believe that we have now appropriately explained that while metabolites present in both cytosol and mitochondria cannot be distinguished experimentally, we account for this by fitting to the pooled amount. The model can then predict the individual cytosolic and mitochondrial levels by using the relative volumes of the sub-cellular compartments.

Moreover, the conditions in which the comparison is made are completely obscure

We apologize for not making clear the conditions under which the model was simulated. It was certainly not our intention to obscure these details. We have revised the manuscript to address this point.

In the Figure 1 caption, we previously stated: “We compared model predictions at the 60-minute time point to qualitative shifts in metabolism as reported in the literature.” We have now expanded this to mention that the comparisons are shown for the fold change of the 16.7/2.8mM conditions (Line 139). In addition, to be more transparent, we now explicitly mention that the qualitative data for Figure 1 can be seen in Dataset S1. We have also updated the captions for Figures 2 and 3.

No parameters set(s) are defined to clarify how the simulations producing the results presented in each figure where performed.

There are not specific parameter sets defined for each application of the model. Rather, we used a single predictive model with multiple best-fit parameters (this accounts for uncertainty in the estimated parameters). We have added this to Line 359. In addition, as mentioned above, all parameter values are given in the new Supplementary Table S5.

For example:
(a) Does the initial point at 2.8 mM Glucose before the perturbation correspond to a steady state? It should be at steady state when perturbed with 16.7 mM and then it should evolve in time reproducing the experimental data, does it?

We appreciate the reviewer’s point about steady state and agree this is an important point. However, we respectfully submit that this point is not relevant for the conditions under which the model is being simulated.

Our computational simulations are aimed at replicating the experimental conditions used to collect the data. We take as an example the study by Malmgren, where beta cells were stimulated with either 2.8 mM glucose or 16.7 mM glucose for 60 minutes. Their paper reports the fold-change in the levels of 14 metabolites at the 60-minute timepoint. It is not shown in the experimental paper that the pancreatic beta cells cultured *in vitro* reach a steady state prior to treatment with 16.7mM glucose. This is true for the other studies we found as well – there is no indication that a steady state condition was reached before glucose stimulation. Thus, we simulate the model for the same conditions: we run the model for 60 minutes with 2.8 mM glucose; in a separate simulation, we run the model for 60 minutes with 16.7 mM glucose. Then we take the ratio of the predicted metabolite concentrations at the 60 minute timepoint.

We cannot state with certainty that it is reasonable to expect the cultured cells to reach a steady state during the short glucose treatment period. Therefore, we did not make a steady-state analysis a central part of the paper. We have properly shown that the model is mathematically sound: we provide the stoichiometric matrix and calculate conserved metabolite pools, show that the model does reach a steady state condition for a relevant glucose concentration (16.7 mM), and demonstrate the fitted model is in close agreement with data used for training and with separate validation datasets.

We believe that we have appropriately applied the model, in a way that aligns with experimental studies.

(b) Moreover, the whole parametric set should be provided either in the main text or the supplemental information for a reader to be able to produce the results.

We very much agree and thank the reviewer for this point. The revised manuscript now lists all model parameters, and their sources, in the new Supplemental Table S5. This makes it easier for the reader to see all the parameters (including the best-fit parameter sets) in one place, rather than having them search the model code on GitHub or look at the plot of the fitted parameters in Figure S3.

The figure legends are incomplete since the conditions in which the results shown were obtained are not stated.

We thank the reviewer for raising this point. We have now added more detail in the captions for Figures 1, 2, and 3, to explicitly mention the extracellular glucose conditions simulated.

The data in Figure 4 is specially challenging. The data about the fluxes show that most metabolites are far from a steady state, e.g. 100 (Units?) of glucose is being provided but only 25.1 glucose are consumed. About 15 (what?) of pyruvate are being consumed while less than 6 are entering the mitochondrial pyruvate pool. This way of presenting the fluxes is confusing and unorthodox, to say the least, mainly without the clarification of the conditions represented by these completely imbalanced fluxes.

We appreciate the reviewer’s point, and we have updated the figure as detailed below.

First, we note that it is fairly common to show flux values relative to a chosen import reaction (for example, glucose)^1,4,5,7^. However, we agree that we could better clarify this point. We have revised the manuscript to add more detail clarifying that the values shown are a percentage of the glucose import flux. The model calculates fluxes as mM/minute. When reporting the fluxes scaled to glucose import in Figure 4, the flux values shown are unitless. This has been adjusted in the figure caption (Lines 566-574).

In the previous version, Figure 4 showed the dynamic metabolic fluxes (relative to glucose uptake), averaged over the 60-minute glucose stimulation (16.7 mM). Our goal was to complement the dynamics of the experimentally-observed metabolite levels shown during the first 60 minutes of treatment in Figure 3. Because the metabolite fold changes were changing during the 60-minute glucose treatment, the metabolic fluxes would not show a steady state distribution, and the flux into a node will not necessarily equal the flux out of a node.

However, we agree with the reviewer that it was not an intuitive presentation of the metabolic fluxes. We have changed Figure 4 to now show the distribution of fluxes at steady state condition, following stimulation with 16.7 mM glucose. We still mirror the simulation conditions used in Figure 3, but now present the fluxes at a later time point, once the system converges to a stable condition. The reviewer will now see that in the updated version of Figure 4, the fluxes into a metabolite node equal the fluxes out, and total flux into the system equals the sum of fluxes for transport reactions out of the system.

We believe these changes eliminate confusion.

In summary, I see that the authors have put a lot of emphasis in the statistical aspects of the modeling data while disregarding a correct presentation of the model with which they have performed that statistical analysis.

We appreciate the reviewer’s time and effort spent to evaluate our work. We believe we have addressed the points about presentation of the results and that all figures reflect the rigor of our computational modeling approach.

**Reviewer #3:**

The authors have addressed all of our previous comments. We look forward to seeing this work published.

[1] L. M. Blank, L. Kuepfer, and U. Sauer, “Large-scale 13C-flux analysis reveals mechanistic principles of metabolic network robustness to null mutations in yeast,” *Genome Biology*, vol. 6, no. 6, p. R49, May 2005, doi: [10.1186/gb-2005-6-6-r49](https://doi.org/10.1186/gb-2005-6-6-r49).

[2] F. P. Guengerich, “Kinetic Modeling of Steady-State Situations in Cytochrome P450 Enzyme Reactions,” *Drug Metab Dispos*, vol. 47, no. 11, pp. 1232–1239, Nov. 2019, doi: [10.1124/dmd.119.088732](https://doi.org/10.1124/dmd.119.088732).

[3] N. Jahan, K. Maeda, Y. Matsuoka, Y. Sugimoto, and H. Kurata, “Development of an accurate kinetic model for the central carbon metabolism of Escherichia coli,” *Microbial Cell Factories*, vol. 15, no. 1, p. 112, Jun. 2016, doi: [10.1186/s12934-016-0511-x](https://doi.org/10.1186/s12934-016-0511-x).

[4] K. Maeda, N. Okahashi, Y. Toya, F. Matsuda, and H. Shimizu, “Investigation of useful carbon tracers for 13C-metabolic flux analysis of Escherichia coli by considering five experimentally determined flux distributions,” *Metabolic Engineering Communications*, vol. 3, pp. 187–195, Dec. 2016, doi: [10.1016/j.meteno.2016.06.001](https://doi.org/10.1016/j.meteno.2016.06.001).

[5] J. M. Park, T. Y. Kim, and S. Y. Lee, “Prediction of metabolic fluxes by incorporating genomic context and flux-converging pattern analyses,” *Proceedings of the National Academy of Sciences*, vol. 107, no. 33, pp. 14931–14936, Aug. 2010, doi: [10.1073/pnas.1003740107](https://doi.org/10.1073/pnas.1003740107).

[6] M. Roy and S. D. Finley, “Computational Model Predicts the Effects of Targeting Cellular Metabolism in Pancreatic Cancer,” *Front Physiol*, vol. 8, p. 217, 2017, doi: [10.3389/fphys.2017.00217](https://doi.org/10.3389/fphys.2017.00217).

[7] J. A. Wushensky, T. Youngster, C. M. Mendonca, and L. Aristilde, “Flux Connections Between Gluconate Pathway, Glycolysis, and Pentose–Phosphate Pathway During Carbohydrate Metabolism in Bacillus megaterium QM B1551,” *Frontiers in Microbiology*, vol. 9, 2018, Accessed: Aug. 18, 2022. [Online]. Available: <https://www.frontiersin.org/articles/10.3389/fmicb.2018.02789>
